# A Meta-Analysis of the Metabolic Syndrome Prevalence in the Global HIV-Infected Population

**DOI:** 10.1371/journal.pone.0150970

**Published:** 2016-03-23

**Authors:** Kim A. Nguyen, Nasheeta Peer, Edward J. Mills, Andre P. Kengne

**Affiliations:** 1 Non-Communicable Diseases Research Unit, South African Medical Research Council, Cape Town, South Africa; 2 Department of Medicine, University of Cape Town, Cape Town, South Africa; 3 Global Evaluation Science, Vancouver, Canada; Centro de Biología Molecular Severo Ochoa (CSIC-UAM), SPAIN

## Abstract

**Background:**

Cardio-metabolic risk factors are of increasing concern in HIV-infected individuals, particularly with the advent of antiretroviral therapy (ART) and the subsequent rise in longevity. However, the prevalence of cardio-metabolic abnormalities in this population and the differential contribution, if any, of HIV specific factors to their distribution, are poorly understood. Therefore, we conducted a systematic review and meta-analysis to estimate the global prevalence of metabolic syndrome (MS) in HIV-infected populations, its variation by the different diagnostic criteria, severity of HIV infection, ART used and other major predictive characteristics.

**Methods:**

We performed a comprehensive search on major databases for original research articles published between 1998 and 2015. The pooled overall prevalence as well as by specific groups and subgroups were computed using random effects models.

**Results:**

A total of 65 studies across five continents comprising 55094 HIV-infected participants aged 17–73 years (median age 41 years) were included in the final meta-analysis. The overall prevalence of MS according to the following criteria were: ATPIII-2001:16.7% (95%CI: 14.6–18.8), IDF-2005: 18% (95%CI: 14.0–22.4), ATPIII-2004-2005: 24.6% (95%CI: 20.6–28.8), Modified ATPIII-2005: 27.9% (95%CI: 6.7–56.5), JIS-2009: 29.6% (95%CI: 22.9–36.8), and EGIR: 31.3% (95%CI: 26.8–36.0). By some MS criteria, the prevalence was significantly higher in women than in men (IDF-2005: 23.2% vs. 13.4, p = 0.030), in ART compared to non-ART users (ATPIII-2001: 18.4% vs. 11.8%, p = 0.001), and varied significantly by participant age, duration of HIV diagnosis, severity of infection, non-nucleoside reverse transcriptase inhibitors (NNRTIs) use and date of study publication. Across criteria, there were significant differences in MS prevalence by sub-groups such as in men, the Americas, older publications, regional studies, younger adults, smokers, ART-naïve participants, NNRTIs users, participants with shorter duration of diagnosed infection and across the spectrum of HIV severity. Substantial heterogeneities across and within criteria were not fully explained by major study characteristics, while evidence of publication bias was marginal.

**Conclusions:**

The similar range of MS prevalence in the HIV-infected and general populations highlights the common drivers of this condition. Thus, cardio-metabolic assessments need to be routinely included in the holistic management of the HIV-infected individual. Management strategies recommended for MS in the general population will likely provide similar benefits in the HIV-infected.

## Introduction

The Global Burden of Disease Expert Group estimated that approximately 30 million people were infected with HIV worldwide in 2013, the majority of whom reside in sub-Saharan Africa [1]. Life expectancy and quality of life in those infected with HIV have improved dramatically with the introduction of effective antiretroviral therapy (ART). Between 1990 and 2013, ART saved an estimated 19.1 million life-years in HIV-infected adults [1].

With increased longevity in HIV-infected individuals other diseases are likely to develop, similar to the general population. These include obesity, type 2 diabetes mellitus (T2DM) and other cardio-metabolic diseases. Although exposure to risky behaviours of unhealthy diets and reduced physical activity levels contribute to these conditions [2], additional influences unique to HIV-infected populations further increase their susceptibility to cardio-metabolic abnormalities. For example, the use of ART is associated with body fat redistribution and cardio-metabolic abnormalities such as hypertension, dyslipidaemia, insulin resistance, and dysglycaemia [3]. Moreover, HIV infection itself through chronic inflammation and immune dysfunction mechanisms is assumed to be an important determinant of dyslipidaemia, atherosclerosis and T2DM [4].

Cardio-metabolic abnormalities frequently cluster and manifest as the metabolic syndrome (MS), a constellation of interrelated metabolic disorders comprising abdominal obesity, raised blood pressure, dyslipidaemia and hyperglycaemia. The importance of the MS is that it is a powerful predictor of future cardiovascular disease and T2DM [5]. Therefore, determining the magnitude of MS in a given population highlights the need for preventive and management strategies, and enables healthcare services planning.

This is particularly relevant in HIV-infected populations who have the potential to develop cardio-metabolic abnormalities and MS through multiple pathways. Notably, the prevalence of MS in HIV-infected populations and the differential contributions, if any, of HIV specific influences on the estimates have yet to be fully examined. Accordingly, we conducted a systematic review and meta-analysis to assess the MS prevalence and its relationship with HIV specific characteristics in the global HIV-infected population.

## Methods

### Identification of relevant studies

We undertook a comprehensive electronic search across major databases including Medline, CINAHL, Academic Search Premier, Africa-Wide Information and Scopus to identify relevant studies. The search terms comprised combinations of MESH terms, CINAHL headings, and free words relating to prevalence, metabolic syndrome, and HIV/AIDS (S1 Table). Additionally, we traced the citations of identified articles via the ISI Web of Knowledge, and scanned the reference lists of review papers and conference proceedings. We also examined publications on the websites of key organisations such as UNAIDS, WHO, and International AIDS Society. We limited the search to studies reported from January 1998 to April 2015 because highly active antiretroviral therapy (HAART) was introduced only in 1996 [6] and the first MS criteria were defined in 1998 [7]. The last search data was 30th April 2015.

### Selection of included studies

**Fig 1:**
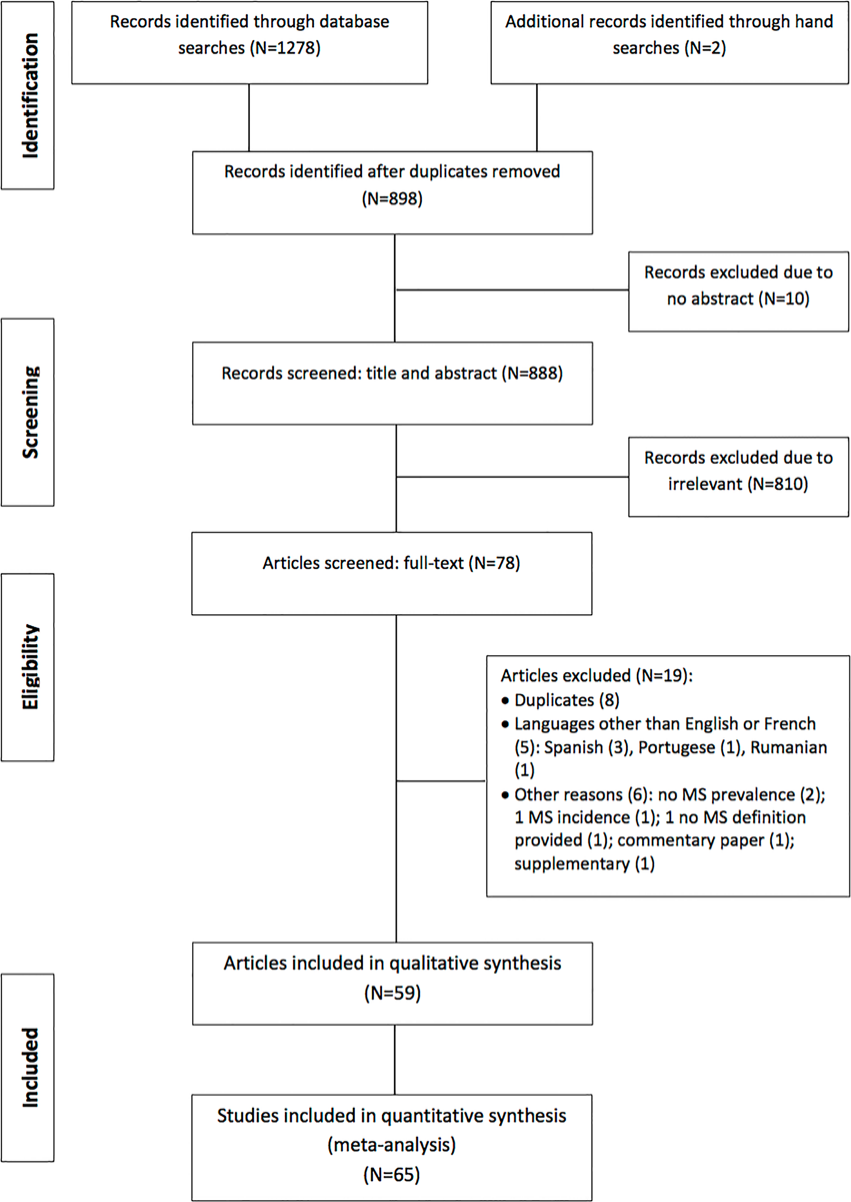
Flow diagram for the selection of studies.

Two investigators (KAN and NP) independently reviewed the studies by title, abstract and full text where relevant for inclusion. Disagreements were resolved by consensus or by consulting a third investigator (APK). Included studies had to: 1) be population- or hospital-based cross-sectional studies, 2) comprise adults diagnosed with HIV-infection, treated or not; 3) report the prevalence of MS overall and by different subgroups of interest, according to any of the internationally accepted diagnostic criteria for MS (S3 Table); or provide enough data to estimate this prevalence; and 4) be published in English or French. We made no restriction by sample size, sampling methods or study setting. For studies reported more than once, the article with the largest number of participants was used. If an article reported multiple surveys conducted in different countries, each survey was counted as a separate study **(Fig 1)**.

### Assessment of the methodological quality of included studies

We evaluated the methodological quality of the included studies using a checklist adapted from Hoy et al. [8]. It consists of nine questions that assesses the representativeness of the sample, the sampling technique, the response rate, the data collection method, the measurement tools, the case definitions, and the statistical reporting. Each checked question was scored either as “1” or “0” corresponding respectively to “low risk of bias” and “high risk of bias”. The total score ranged from 0 to 9 with the overall score categorised as follows: 7 to 9: “low risk of bias”, 4 to 6: “moderate risk”, and 0 to 3: “high risk” (S2 Table). For each included study, we also estimated the precision (C) or margin of error, considering the sample size (SS) and the observed prevalence (p) of MS from the formula SS = Z^2^*p*(1-p)/C^2^ where Z was the z-value fixed at 1.96 across studies (corresponding to 95% confidence interval). The desirable margin of error was 5% (0.05) or lower.

### Data extraction

Relevant data for this review were extracted using a purposeful design and a piloted extraction form. The information extracted included 1) Author details [names and year of publication]; 2) Study characteristics [country, study design, setting, data source, sampling method, sample size, data collection period, response rate]; 3) Participants’ characteristics [age, gender, lifestyle habits (smoking, alcohol misuse), HIV-related factors [time since diagnosis, severity of the disease, ART regimens and duration of treatment]; and 4) MS characteristics [diagnostic criteria used, prevalence, number of participants tested and diagnosed with MS overall and by subgroups of interest].

### Data synthesis and analysis

For each included study, the unadjusted prevalence of MS was estimated (number with MS/total number of participants tested) overall and across major subgroups of interest. We used DerSimonian-Laird random effects models to combine estimates from different studies to generate the overall prevalence of MS according to each diagnostic criteria. The random effects model was chosen over the fixed effects in anticipation of substantial variations in MS prevalence estimates across the included studies. To minimise the effect of extreme prevalence on the overall estimates, we first stabilised the variance of the raw prevalence with a single arcsine transformation before pooling the data [9].

To account for the small number of studies that applied some definition criteria, and also to account for similarities between some criteria, a decision was made to group together studies that applied the Adult Treatment Panel III (ATPIII)-2004 and ATPIII-2005 criteria into the ATPIII-2004-2005 group. Furthermore, studies that applied variants of the same criteria (e.g. through the substitution of variables) were assessed together with studies that applied the original criteria.

We assessed the heterogeneity between studies using the Cochran’s Q, I^2^ and H statistics [10]. Noteworthy is that statistical approaches to assess heterogeneity can yield spurious results within uncontrolled studies [11]. We explored the sources of heterogeneity by comparing the prevalence of MS between subgroups defined by naturally occurring categories (e.g. gender and geographic regions), or by using median values of the summary estimates of the continuous characteristics (e.g. age, ART duration) across all eligible studies. Subgroups comparisons then used the Q-test based on the Analysis of the Variance (ANOVA). Publication bias was assessed using the funnel plots supplemented by formal statistical assessments using the Egger test of bias [12]. All analyses were performed using the R programme (version 3.0.3 [2014-03-06]) and “*meta*” package.

The following sections report the findings of the systematic review and meta-analyses. The data are presented by the overall MS prevalence as well as by subgroups of age, gender, HIV-related factors, study location, publication year, sample size, and smoking status. Within each subgroup, MS prevalence is presented by the definition criteria used.

## Results

### The review process

The process for selecting the relevant studies is summarised in Fig 1. In total, 1280 records were identified via database searches. After removing all duplicates, we scanned the titles and abstracts of 888 articles, of which 78 articles were further reviewed via full-texts. Of these, 59 articles met the inclusion criteria and were selected for this review. One article reported surveys conducted in seven South American countries, leading to a total of 65 studies in the main analyses.

### Methodological quality of included studies

In all, 18 studies were categorised as having a low risk of bias while the remainder had a moderate risk of bias. However, 37 studies did not indicate how participants were selected; seven studies reported some form of random selection whereas 21 studies indicated a non-random sampling technique. A total of 25 studies reported the response rates which ranged from 31.2% to 100% (median 88%).

### Characteristics of included studies

The characteristics of the included studies are summarised in Table 1. Studies from all continents were represented as follows: Europe: 23, the Americas: 26, Africa: nine and Asia: four, while three studies were intercontinental. Of the 65 included studies, 33 were localised studies, mainly conducted in urban settings, while the rest had national coverage. With regards to the actual study sites, the majority (58) were solely hospital- or clinic-based, four were community-based and three studies involved both locales. While about half of the studies (34) collected data before or during 2007, only one-fifth (12 studies) were published in the same period.

**Table 1:** Characteristics of the studies included in the review.

Reference	Publication year	Country	Area	Study site	Study type	Study period	Sampling	Sample size	Response rate (%)	Mean age (years)	Selection criteria	Quality grade * (Risk)	Precision (margin of error)
**Intercontinental**													
Samaras, et al [25]	2007	USA, Europe, Australia, Asia, South America	National	Hospital + community	C/C	Not provided	Unspecified	788	NR	-	Age ≥ 17 years; not diagnosed with AIDS	Low	0.03
Wand, et al [28]	2007	Australia, Brazil, Canada, New Zealand, 17 Europrean countries	Urban	Hospital	C/S	1999–2002	Random	881	94	38.7	Adults not receiving ART	Low	0.02
Worm, et al [52]	2010	USA, Australia, 21 European countries	National	Hospital (212 clinics)	C/S	2006–2007	Not random	23852	NR	38	Adults on ART and regular follow-up	Low	0.01
**Americas**													
Baum, et al [16]	2006	USA	National	Community	C/S	2002–2003	Unspecified	118	NR	41.7	Adult chronic drug users	Moderate	0.06
Jacobson, et al [37]	2006	USA	Urban	Community	C/S	2000–2003	Unspecified	477	NR	-	Self-selected	Moderate	0.04
Johnsen, et al [27]	2006	USA	National	Hospital	C/C	2002–2003	Unspecified	97	NR	41	Women with BMI≥20 kg/m^2^ and only on ART chronic medications	Moderate	0.09
Mondy, et al [44]	2007	USA	Urban	Hospital	C/S	2005	Not random	471	78	-	All clinic attendees during the study period	Moderate	0.04
Adeyemi, et al [60]	2008	USA	Urban	Hospital	C/S	2005–2006	Unspecified	121	NR	54	Age ≥ 50 years; outpatient	Moderate	0.09
Sobieszczyk, et al [32]	2008	USA	Urban	Hospital + community	C/S	2000–2004	Unspecified	1725	NR	40	Women	Low	0.02
Sterling, et al [18]	2008	USA	Urban	Hospital	C/S	1998–2006	Unspecified	222	82	45.4	Adults co-infected with HCV	Moderate	0.04
Ances, et al [61]	2009	USA	National	Hospital	C/C	Not provided	Unspecified	66	NR	41	Cryptogenic stroke (case subgroup)	Moderate	0.09
Pullinger, et al [24]	2010	USA	Urban	Community	C/S	2005–2007	Unspecified	296	84.6	45.3	Age ≥ 18 years; diagnosed duration ≥3 months	Moderate	0.05
Krishnan, et al [38]	2012	USA	National	Hospital	C/S	2001–2007	random	2247	88	-	Age ≥13 years	Low	0.02
Hadigan, et al [62]	2013	USA	Urban	Hospital (2 clinics)	C/S	2007–2011	Not random	182	72	45	Absence of chronic NCDs or co-infection	Moderate	0.05
Tiozzo, et al [63]	2015	USA	Urban	Hospital	C/S	2013	Not random	89	90	48	Age ≥18 years on ART	Moderate	0.1
Da Silva, et al [64]	2009	Brazil	Urban	Hospital (7 centres)	C/S	2004–2006	Not random	319	NR	39.5	ART use ≥ 2 months, and no anti- lipid agents	Moderate	0.04
Cahn, et al [23]	2010	7 Latin American countries	National	Hospital (61 centres)	C/S	2006–2007	Unspecified	4010	NR	41.9	ART use ≥1 month	Moderate	0.01
Leite, et al [30]	2010	Brazil	Urban	Hospital	C/S	2008	Unspecified	100	NR	-	-	Moderate	0.1
Ramirez-Marrero, et al [20]	2010	Puerto Rico	Urban	Hospital + community	C/S	2003–2007	Random	897	31.2	44.7	-	Low	0.03
Lauda, et al [65]	2011	Brazil	Urban	Hospital	C/S	2007–2008	Unspecified	249	NR	-	Age ≥18 years	Moderate	0.05
Alencastro, et al [66]	2012	Brazil	Urban	Hospital	C/S	Not provided	Not random	1240	96	38.6	Age 18–79 years	Low	0.02
Gasparotto, et al [67]	2012	Brazil	National	Hospital (multiple-centres)	C/S	Not provided	Unspecified	614	NR	42.6	Age ≥18 years; ART use ≥1 year; viral load ≤50 copies/ml	Moderate	0.04
Signorini, et al [43]	2012	Brazil	National	Hospital	C/S	2005	Unspecified	819	NR	41	Age ≥18 years	Low	0.03
**Europe**									NR				
Gazzaruso, et al [68]	2003	Italy	National	Hospital	C/S	Not provided	Unspecified	287	NR	41	ART use	Moderate	0.05
Jerico, et al [19]	2005	Spain	Urban	Hospital	C/S	2003	Unspecified	710	88	41.9	Age ≥20 years; no evidence of AIDS or ART disruption	Low	0.03
Bergersen, et al [69]	2006	Norway	Urban	Hospital	C/S	2000–2001	Not random	263	78	43.3	-	Moderate	0.04
Estrada, et al [70]	2006	Spain	National	Hospital	C/S	Not provided	Not random	146	NR	40.6	ART use ≥6 months, no active opportunistic affection	Moderate	0.06
Bonfanti, et al [71]	2007	Italy	Urban	Hospital (18 centers)	C/S	2005	Not random	1243	98.4	43.2	-	Moderate	0.02
Palacios, et al [51]	2007	Spain	National	Hospital	C/S	2002–2003	Unspecified	60	81	40.9	ART use ≥48 weeks	Moderate	0.09
Badiou, et al [49]	2008	France	National	Hospital	C/S	1999	Not random	232	NR	41	-	Low	0.04
Martin, et al (SHIVA study) [26]	2008	France	Urban	Hospital	C/S	2003	Unspecified	140	86.9	-	-	Moderate	0.04
Schillaci, et al [72]	2008	Italy	Urban	Hospital	C/C	Not provided	Unspecified	39	NR	37	Outpatients; no ART	Moderate	0.12
Hansen, et al [73]	2009	Denmark	National	Hospital	C/S	2004–2006	Unspecified	566	75.7	44.1	Age ≥18 years	Low	0.04
Young, et al [74]	2009	Switzerland	National	Hospital	C/S	2000–2006	Unspecified	1644	70	-	ART use	Low	0.02
Bonfanti, et al [50]	2010	Italy	Urban	Hospital (14 centers)	C/S	2007	Not random	292	NR	37	Age ≥18 years; ART naive	Moderate	0.04
Calza, et al [75]	2011	Italy	Urban	Hospital	C/S	2009	Not random	755	NR	37	Outpatients	Moderate	0.02
Cubero, et al [42]	2011	Spain	National	Hospital	C/S	Not provided	Not random	159	NR	39	1^st^ line ART regimen, no kidney or liver disease, no lipid modifying treatment or hormone use	Moderate	0.07
Elgalib, et al [41]	2011	UK	Urban; Peri-urban	Hospital (2 centers)	C/S	2005–2006	Random	678	66.4	39.5	-	Low	0.03
Freitas, et al [29]	2011	Portugal	National	Hospital	C/S	Not provided	Unspecified	345	NR	43.8	ART use lipodystrophy	Moderate	0.05
Guaraldi, et al [76]	2011	Italy	National	Hospital (2 centers)	C/S	2007–2008	Unspecified	103	NR	46.9	Age ≥18 years on ART	Moderate	0.06
Janiszewski et al [77]	2011	Italy	National	Hospital	C/S	2005–2009	Unspecified	2322	NR	-	ART use ≥ 18 months	Moderate	0.02
Biron, et al [47]	2012	France	National	Hospital (5 centers)	C/S	2000–2007	Not random	269	85.7	43	Aged ≥18 years, ART use for 1–4 years without disruption	Low	0.05
Guaraldi, et al [31]	2012	Italy	National	Hospital (2 centers)	C/S	2009–2010	Unspecified	133	NR	-	Men, sexually active in the 4 last weeks	Moderate	0.07
Maloberti, et al [54]	2013	Italy	National	Hospital	C/S	Not provided	Unspecified	108	NR	-	Free of known CVD risk factors	Moderate	0.07
De Socio, et al (HIV-Hy study) [21]	2014	Italy	National	Hospital	C/S	2010–2011	Not random	765	93	45.6	-	Moderate	0.03
Sawadogo, et al [22]	2014	Burkina Faso	Urban	Hospital	C/S	2011	Random	400	NR	41.4	Age ≥18 years; ART use ≥ 6 months	Moderate	0.03
**Africa**													
Zannou, et al [17]	2009	Benin	Urban	Hospital	C/S	2004–2005	Unspecified	79	90	38	Age ≥ 16 years; ART use; not obese	Moderate	0.07
Awotedu, et al [13]	2010	South Africa	Urban	Hospital	C/S	2009–2010	Not random	196	NR	36.8	No lipid modifying medications	Moderate	0.07
Fourie, et al [78]	2010	South Africa	Urban; Rural	Community	C/S	2005	Random	300	NR	44	Aged ≥35 years; no chronic medications or self-reported disease	Moderate	0.05
Ayodele, et al [48]	2012	Nigeria	Urban	Hospital	C/S	Not provided	Not random	291	94	39.5	No liver or thyroid disease or concurrent infections	Moderate	0.05
Berhane, et al [79]	2012	Ethiopia	Urban	Hospital	C/S	2010	Not random	313	100	-	Age ≥18 years, ART use ≥6 weeks	Moderate	0.05
Muhammad, et al [80]	2013	Nigeria	Urban	Hospital	C/S	2009	Not random	200	NR	32.5	Age ≥18 years; not diagnosed with hypertension, diabetes or dyslipidaemia before commencing ART	Moderate	0.05
Ngatchou, et al [15]	2013	Cameroon	Urban	Hospital	C/S	2009–2010	Not random	108	NR	39	ART-naïve adults; no documented diabetes, hypertension or dyslipidaemia	Moderate	0.09
Mbunkah, et al [46]	2014	Cameroon	National	Hospital	C/S	2010–2011	Unspecified	173	100	38.7	-	Low	0.05
Tesfaye, et al [45]	2014	Ethiopia	Urban	Hospital	C/S	2013	Random	374	97.2	32.6	Age ≥18 years	Low	0.04
**Asia**													
Gupta, et al [40]	2011	India	Urban	Hospital	C/S	2007–2009	Not random	68	NR	35.9	ART-naïve; no chronic medications	Moderate	0.1
Wu, et al [14]	2012	Taiwan	National	Hospital	C/S	2008–2009	Unspecified	803	60.2	-	Age ≥18 years	Low	0.03
Bajaj, et al [81]	2013	India	Urban	Hospital	C/S	2010–2011	Not random	70	NR	-	No comorbid diabetes or hypertension	Moderate	0.09
Jantarapakde, et al [82]	2014	Thailand	National	Hospital (6 centres)	C/S	2009–2011	Unspecified	580	99	37	Adults	Low	0.03

BMI, body mass index; C/C, case-control; C/S, cross-sectional; HCV, hepatitis C virus; NCDs, non-communicable diseases; NR, not reported.

*Quality grades: Low risk (score range, 7–9), Moderate risk (score range, 4–6), and High risk (score range, 0–3).

The studies consisted of 39 to 23853 participants with men comprising 19–95% (median 70.7%) of the samples [13,14]. The median age of participants was 41 years (range 17–73 years). Smoking prevalence, reported in 47 studies, was 0–84% (median 39.8%) [15,16]. In the 37 studies with data on CD4 cell count, levels ranged from 105 cells/μL (Benin) [17] to 535 cells/μL (USA) [18] (median 394 cells/μL). The timespan of diagnosed HIV infection, reported in 20 studies, was 19.3 to 224.4 months [15,18] (median 67.6 months) while the duration of ART, described in 21 studies, was 14.6–78 months [17,19], (median 27 months). In the 28 studies that reported on ART usage, 45–94% of the HIV-infected participants, were on ART [20,21] (median 76.2%). Of those on ART, 17.3–61.5% (median 37.4%) were on protease inhibitors (PIs) [22,23], 19.4% (median 43.4%) on non-nucleoside reverse transcriptase inhibitors (NNRTIs) [22,24], and 1.5–85.5% (median 77.0%) on nucleoside reverse transcriptase inhibitors (NRTIs) [21,25]. Very few studies provided information on the stage of the disease (S4 Table).

The included studies applied various international criteria to diagnose MS (S5 Table). Fifty-one studies applied a single set of criteria; the most frequently used was the ATPIII-2001 in 30 studies followed by the ATPIII-2004-2005 (14 studies). The International Diabetes Federation (IDF)-2005, Joint Interim Statement (JIS)-2009 and modified ATPIII-2005 criteria were used in 2 studies each while the European Group for the Study of Insulin Resistance (EGIR)-2003 in one only. Of the 14 studies that compared the MS prevalence using two or more criteria, the following combinations were reported: two criteria: IDF-2005 + ATPIII-2001 (7 studies), and IDF-2005 + ATPIII-2005 (4 studies), and three criteria (1 study each): IDF-2005 + ATPIII-2005 + JIS-2009; IDF-2005 + ATPIII-2001 + EGIR-2003; IDF-2005 + ATPIII-2001 + EGIR-2003.

### Overall prevalence of metabolic syndrome

**Fig 2:**
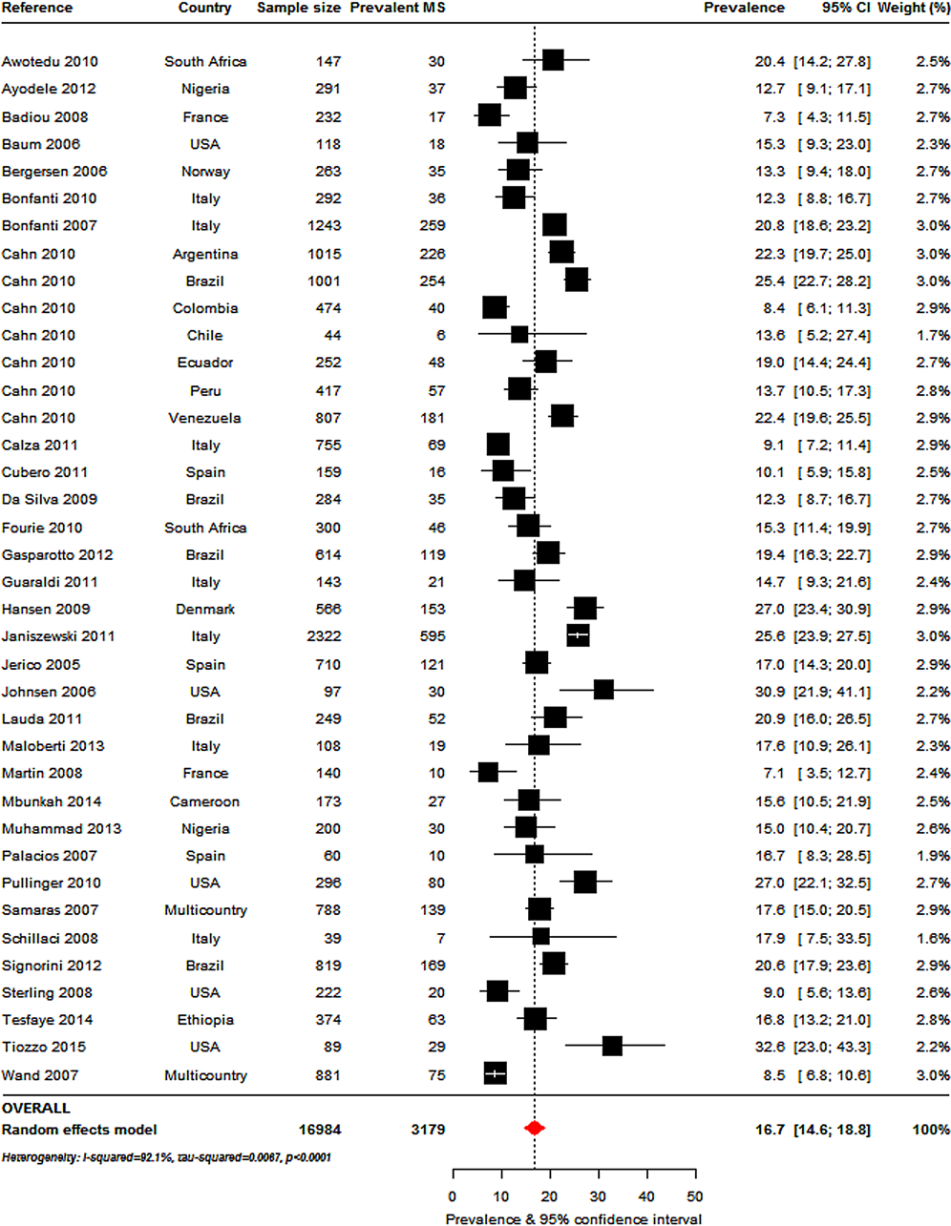
Overall metabolic syndrome prevalence in the HIV-infected: Adult Treatment Panel III (ATPIII) 2001 criteria. For each study the black box represents the study estimate (prevalence of metabolic syndrome [MS]) and the horizontal bar about the 95% confidence intervals (95%CI). The size of the boxes is proportional to the inverse variance. The diamond at the lower tail of the figure is for the pooled effect estimates from random effects models. The proportional contribution of each study (weight) to the pooled estimates is also shown, together with the prevalence estimates and measures of heterogeneity. The dotted vertical line is centred on the pooled estimates.

**Fig 3:**
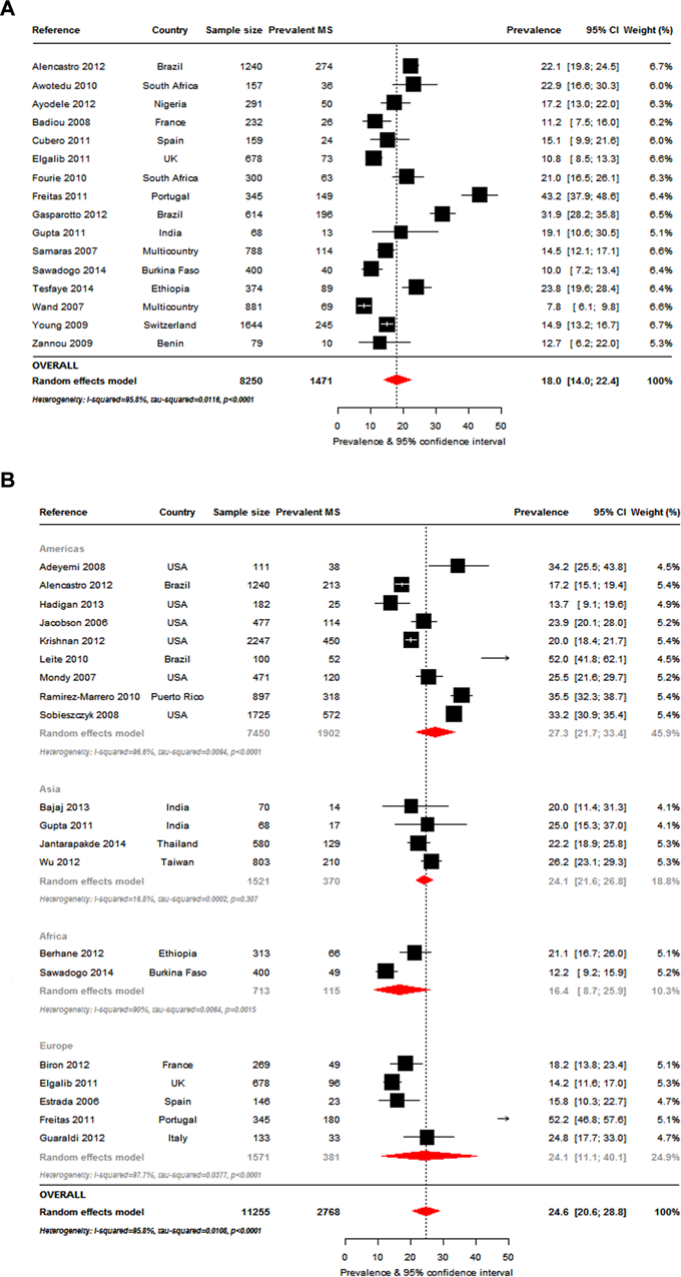
Overall metabolic syndrome prevalence in the HIV-infected. Figure panels are for the prevalence of metabolic syndrome according to the International Diabetes Federation 2005 criteria (panel a), and according to the Adult Treatment Panel III 2005 criteria overall and by continent (panel b). For each study the black box represents the study estimate (prevalence of metabolic syndrome [MS]) and the horizontal bar about the 95% confidence intervals (95%CI). The size of the boxes is proportional to the inverse variance. The diamond at the lower tail of the figure is for the pooled effect estimates from random effects models. The proportional contribution of each study (weight) to the pooled estimates is also shown, together with the prevalence estimates and measures of heterogeneity. The dotted vertical line is centred on the pooled estimates.

The most commonly used criteria to determine MS prevalence, alone or in combination with other criteria, were the ATPIII-2001 (Fig 2: 38 studies, n = 16984), IDF-2005 (Fig 3A: 16 studies, n = 8250) and ATPIII-2004-2005 definitions (Fig 3B: 20 studies, n = 11255). The overall MS prevalence rates by these criteria were 16.7% (95%CI, 14.6–18.8; I^2^ = 92.1%, p-*heterogeneity*<0.001), 18.0% (95%CI: 14.0–22.4; I^2^ = 95.8%, p<0.001) and 24.6% (95%CI: 20.6–28.8; I^2^ = 95.8%, p<0.001), respectively. The prevalence ranges were 7.2% [26] to 31% [27] (ATPIII 2001), 7.8% [28] to 43.2% [29] (IDF 2005) and 12.3% [22] to 52% [30] (ATPIII 2004–2005).

The highest overall MS prevalence was by the EGIR criteria (31.3%, 95%CI: 26.8–36.0; I^2^ = 9.8%, p = 0.300) used in two studies (n = 446) (S1 Fig). A similarly high prevalence by the JIS criteria (29.6%, 95%CI: 22.9–36.8; I^2^ = 91%, p<0.001) was based on four studies (n = 2404) (S2 Fig). MS prevalence by the modified ATPIII 2005 criteria, obtained from two studies (n = 23919), was also high at 27.9% (95%CI: 6.7–56.5; I^2^ = 95.8%, p<0.001) (S3 Fig). However, there were relatively few studies that determined the MS by these three criteria. The margin of error (precision) across studies ranged from 1% to 12%, with only 18 studies (28%) having a margin of error >5% (Table 1).

**Fig 4:**
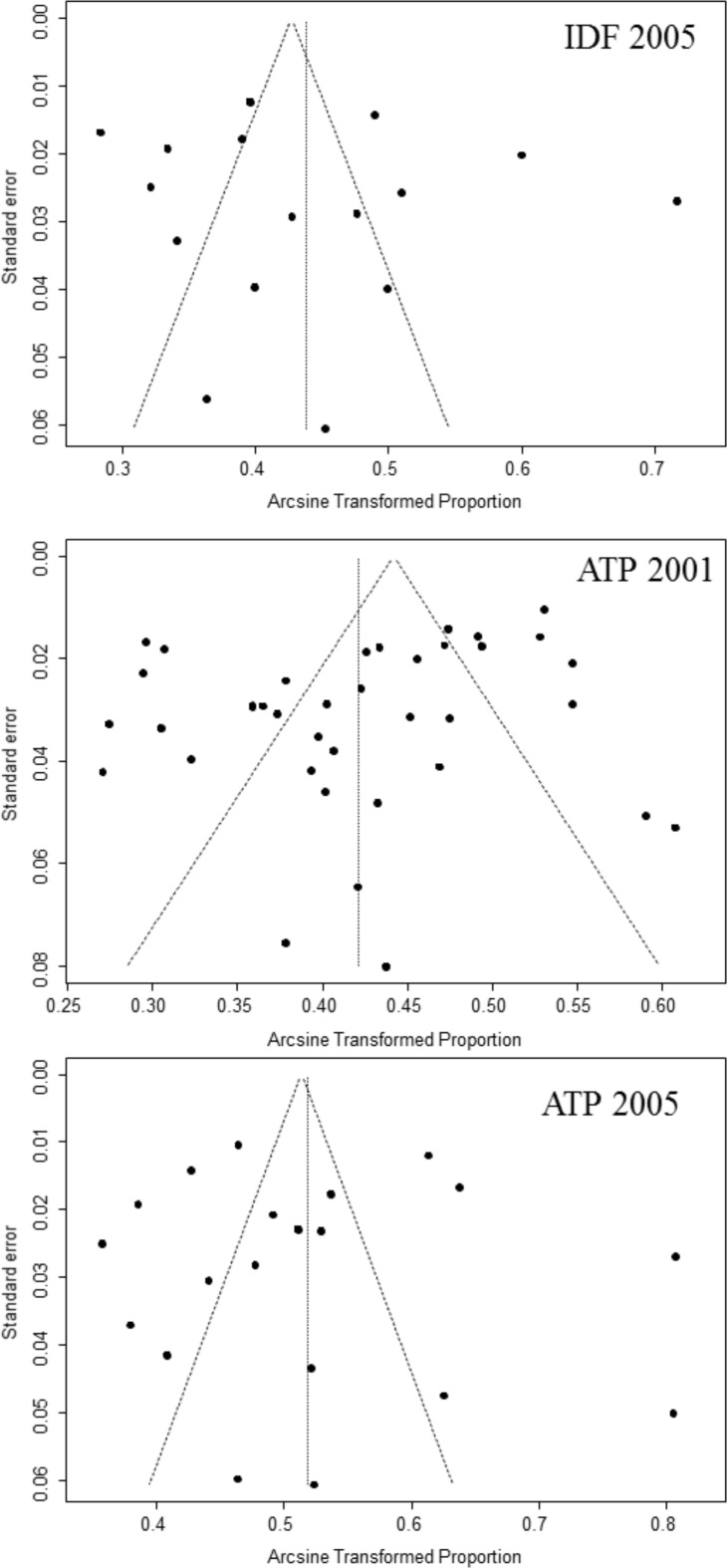
Funnel plots for included studies across different diagnostic criteria for metabolic syndrome. For each diagnostic criteria, the arcsine transformed proportion of participants with metabolic syndrome (relative to the total sample) for each relevant study (horizontal axis) is plotted against its standard error (vertical axis), and represented by the dots. When the dots distribute symmetrically in a funnel shape, this implies an absence of bias. A p-value <0.05 (Egger test) indicates significant publication bias.

With a wide range of 16.7% to 31.3%, the pooled prevalence of MS differed significantly by the various diagnostic criteria (p<0.001). Unsurprisingly, however, MS prevalence by modified criteria was similar to that of studies that used the related original definition (S5 Table). There was insufficient evidence of publication bias (all p ≥0.060 for the Egger test) except for studies that used the ATPIII 2001 criteria (p-Egger test = 0.040; Fig 4)

### Prevalence of metabolic syndrome within and across subgroups

Some of the findings are presented in the accompanying tables and figures while other data such as the prevalence of MS by age, intra-country location, duration of HIV infection, ART status, treatment period and year of publication are reported in the supplementary tables and figures.

#### Age

Older (>median age 41 years) compared with younger participants (≤41years) had higher MS prevalence (S6 Table): ATPIII-2001: 19.7% vs. 13.2%, p<0.001, ATIII 2004–2005: 26.6% vs. 21.5%, p = 0.479 and IDF-2005: 22.3% vs. 16.4%, p = 0.361. Substantial heterogeneity was apparent within age-groups regardless of the criteria (all p-*heterogeneity*<0.001).

#### Gender

**Fig 5:**
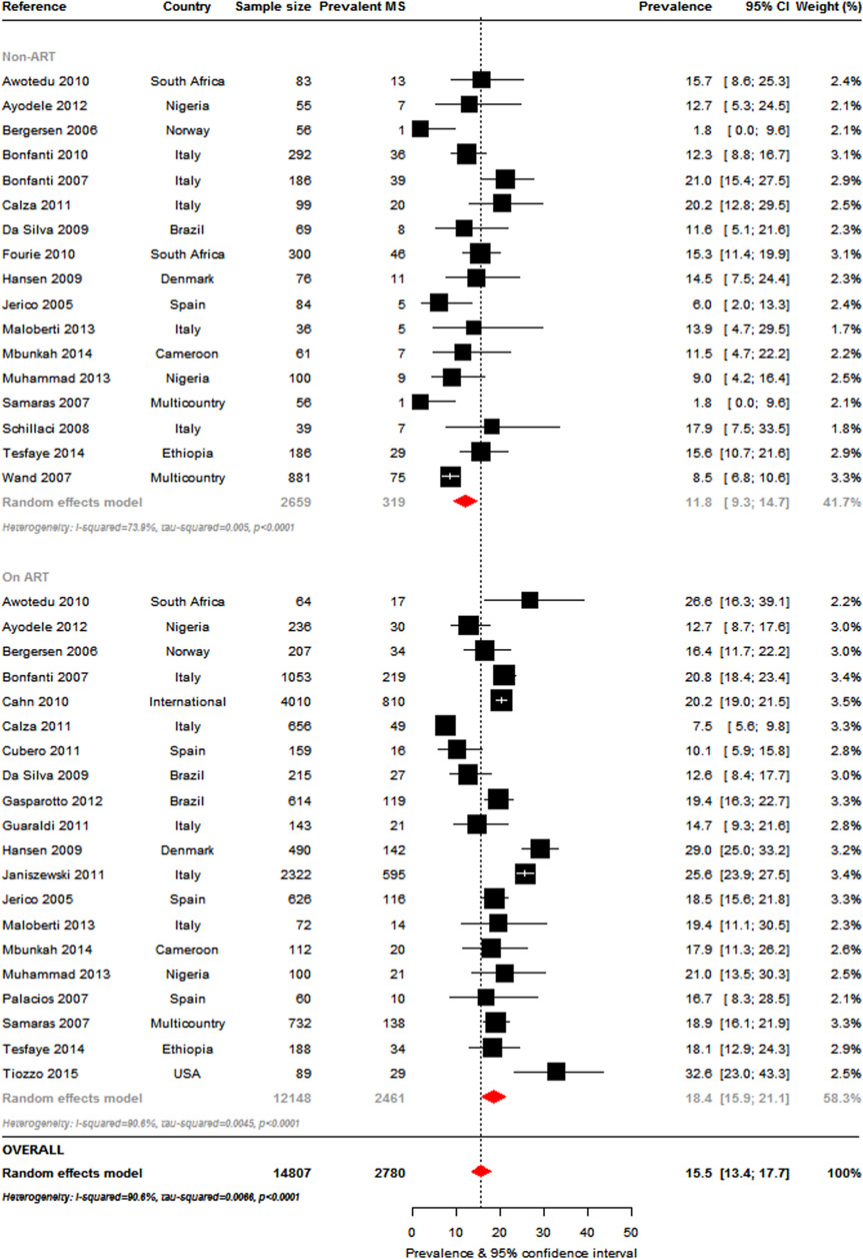
Pooled metabolic syndrome prevalence in the HIV-infected presented by gender: International Diabetes Federation 2005 criteria. For each study the black box represents the study estimate (prevalence of metabolic syndrome [MS]) and the horizontal bar about the 95% confidence intervals (95%CI). The size of the boxes is proportional to the inverse variance. The diamond at the lower tail of the figure is for the pooled effect estimates from random effects models. The proportional contribution of each study (weight) to the pooled estimates is also shown, together with the prevalence estimates and measures of heterogeneity. The dotted vertical line is centred on the pooled estimates. Furthermore, pooled effect estimates are provided separately by gender. The horizontal arrow head indicates that the representation of the effect estimates and 95% confidence intervals has been truncated.

Thirty-two of the 65 studies presented the MS data by gender; of these a single study was conducted in men only (Italy) [31] and two in women only (USA) [27,32]. The criteria most commonly used was the ATPIII 2001 in 16 studies for men (n = 8269) and 17 studies for women (n = 3971). This was followed by the ATPIII 2004–2005 (13 studies, men: n = 5742, women: n = 4470) and IDF 2005 definitions (11 studies, men: n = 3556, women: n = 2293). The MS prevalence in men and women was as follows: ATPIII 2001: 14.6% (95%CI: 11.5–18.1) and 17.5% (95%CI: 14.0–21.2); ATPIII 2004–2005: 23.7% (95%CI: 19.0–28.7) and 26.7% (95%CI: 20.8–33.0); and IDF 2005: 13.4% (95%CI: 8.7–18.9) and 23.2% (95%CI: 15.9–31.4); Fig 5 and S6 Table. MS prevalence by the various criteria was significantly different in men (p = 0.001) but not in women (p = 0.118). Heterogeneity presented within gender-groups across criteria (all p-*heterogeneity*<0.001).

### HIV-related factors

*Duration of diagnosed HIV infection*: The prevalence of MS, categorised by the duration of diagnosed HIV infection (median cut-off point 68 months), differed across MS criteria (S6 Table). By the ATPIII-2001 definition, MS prevalence was significantly higher (p = 0.044) in participants with longer (20.6%, 95%CI: 13.8–28.4) compared to shorter duration of diagnosed HIV-infection (13.2%, 95%CI: 11.2–15.4). However, by the ATPIII-2004-2005, MS prevalence was not significantly different: longer: 32.0% vs. shorter: 19.1%, p = 0.251. There was substantial heterogeneity across and within criteria for MS prevalence by the duration of diagnosed HIV infection; all p-*heterogeneity*<0.030 for within criteria except for studies below the median duration which used the ATPIII-2001 criteria (p-*heterogeneity* = 0.581)

*CD4 counts*: Using a median cut-off point of 394 cells/μL, MS prevalence in participants with high CD4 counts was significantly lower than in those with low CD4 counts by the IDF 2005 criteria: 10.4% (8.2–12.9) vs. 17.5% (14.4–20.8), p<0.001 (S6 Table). However, there was little difference in MS prevalence by CD4 count levels using the ATPIII-2001 (17.4% vs. 15.6%, p = 0.514) and ATPIII-2004-2005 definitions (24.6% vs. 26.5%, p = 0.747). The prevalence across MS diagnostic criteria was significantly different by CD4 count levels (all p≤0.020). Substantial heterogeneity was noted in MS prevalence by CD4 count levels within studies that applied the ATPIII-2001 and ATPIII-2004-2005 criteria (all p-*heterogeneity*≤0.035) but not within studies that used the IDF-2005 definition (p-*heterogeneity*≥0.272).

**Fig 6:**
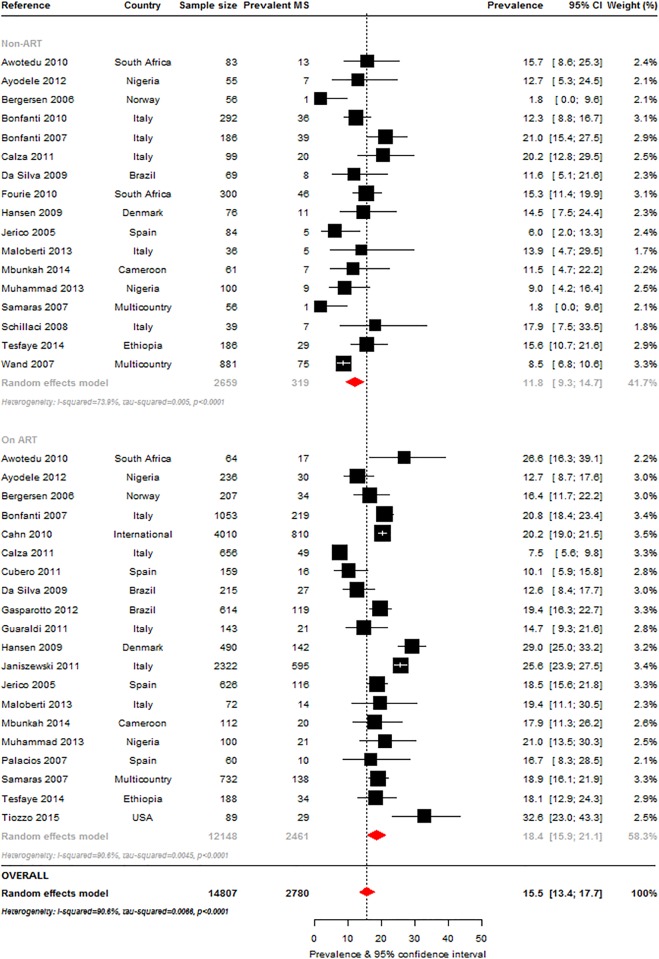
Pooled metabolic syndrome prevalence in the HIV-infected presented by antiretroviral therapy (ART) use: Adult Treatment Panel 2001 criteria. For each study the black box represents the study estimate (prevalence of metabolic syndrome [MS]) and the horizontal bar about the 95% confidence intervals (95%CI). The size of the boxes is proportional to the inverse variance. The diamond at the lower tail of the figure is for the pooled effect estimates from random effects models. The proportional contribution of each study (weight) to the pooled estimates is also shown, together with the prevalence estimates and measures of heterogeneity. The dotted vertical line is centred on the pooled estimates. Furthermore, pooled effect estimates are provided separately by ART use. The horizontal arrow head indicates that the representation of the effect estimates and 95% confidence intervals has been truncated.

*Exposure to antiretroviral therapy*: In studies that included treatment status **(**S6 Table**)**, the most commonly used MS criteria was the ATPIII 2001 (ART-exposed: 20 studies, n = 12148, ART-naïve: 17 studies, n = 2659). MS prevalence, at 18.4% (95% CI: 15.9–21.1) in the ART-exposed, was significantly higher (p = 0.001) than in the ART-naïve (11.8%, 95%CI: 9.3–14.7) (Fig 6). MS prevalence by the IDF 2005 criteria was also higher in the ART-exposed (19.6%, 95%CI: 14.2–25.6) compared to the ART-naïve (14.9%, 95%CI: 8.6–22.6) but this difference was not significant; prevalence was similar by the ATPIII 2004–2005 definition (21.6%, 95%CI: 13.5–31.0 vs. 19.9%, 95%CI: 18.3–21.5). Interestingly, MS prevalence by the various criteria was similar in the ART-exposed (p = 0.730) but significantly different in the ART-naïve (p<0.001). Excluding the non-ART studies based on the ATPIII-2004-2005 where homogeneity was found (p-*heterogeneity* = 0.398), there was significant heterogeneity within ART-exposed and ART-naïve groups (all p-*heterogeneity*<0.001).

*Proportions of ART users and duration of ART treatment*: MS prevalence by a high or low proportion of ART users (median cut-off point 76.2%) was not significantly different within criteria: ATPIII 2001: 15.8% vs. 19.1%, p = 0.172; ATPIII 2004–2005: 38.3% vs. 23.8%, p = 0.256; IDF 2005: 14.4% vs. 19.4%, p = 0.176. Using a cut-off point of 27 months for the median length of ART use, MS prevalence was not significantly different for a longer or shorter treatment duration within MS criteria: ATPIII 2001: 17.1% vs. 15.6%, p = 0.649; ATPIII 2004–2005: 25.6% vs. 14.2%, p = 0.192; IDF 2005: 14.6% vs. 13.4%, p = 0.811. The prevalence within the two sub-groups was similar across MS criteria (all p≥0.111). Substantial heterogeneity was noted within all the above subgroups (all p-*heterogeneity*<0.001).

*ART regimen*: The median proportion of participants using PIs across the included studies was 37.4% (S6 Table). Using this cut-off point, the pooled MS prevalence in studies with more compared to fewer PI users by the IDF 2005 criteria was 18.5% (95%CI: 12.3–25.6) from six studies (n = 4927) versus 10.0% (95%CI: 7.2–13.4) (p = 0.016) in a single study (n = 400). However, the MS prevalence by proportion of PI users was not significantly different by the ATPIII 2001 (17.7% vs. 19.3%, p = 0.593) and ATPIII 2004–2005 definitions (25.8% vs. 30.4%, p = 0.517). MS prevalence, determined by the various criteria, differed across studies of PI users (p = 0.022) and in those with fewer median participants on PIs (p = 0.001).

The median proportion of participants using NNRTIs was 43.4%, the cut-off value used to determine a high and low proportion of users. The pooled MS prevalence in studies with more compared to fewer NNRTI users was 17.2% (95%CI: 9.5–26.6) versus 33.8% (95%CI: 23.4–45.1) (p = 0.020) by the ATPIII 2004–2005 criteria. MS prevalence according to NNRTI regimen status was not significantly different by the ATPIII 2001 (19.5% vs. 15.1%, p = 0.221) and IDF 2005 criteria (10.5% vs. 19.8%, p = 0.058). However, MS prevalence within the two NNRTI subcategories was significantly different by the various MS criteria (all p≤0.007), (S6 Table).

In studies with a high proportion of participants on NRTI (median >77.0%), the pooled prevalence by the ATPIII 2001 criteria was 17.9% (95%CI: 9.4–28.7) compared to 22.8% (95%CI: 14.3–32.7) in studies with fewer participants on NRTIs (p = 0.474) (S6 Table). There was insufficient data by the IDF 2005 and ATPIII 2004–2005 criteria for analysis.

### Study location

*Intercontinental*: The ATPIII 2004–2005 MS criteria was the only one commonly used by studies across continents. The pooled MS prevalence by this criteria was the highest in the Americas (9 studies, n = 7450) at 27.3% (95%CI: 21.7–33.4; I^2^ = 96.6%, p<0.001). This was followed by Europe (5 studies, n = 1571) and Asia (4 studies, n = 1521) where the prevalence was similar. At 24.1% (95%CI: 11.2–40.1; I^2^ = 97.7%, p<0.001) in Europe and 24.1% (95%CI: 21.6–26.8; I^2^ = 16.8%, p = 0.307) in Asia, MS prevalence on these continents were almost as high as in the Americas. MS prevalence in Africa (2 studies, n = 713), however, was much lower at 16.4% (95%CI: 8.7–25.9; I^2^ = 90%, p = 0.002), (Fig 3B and S6 Table). The differences in prevalence across continents was not statistically significant (p = 0.284).

*Intra-country*: MS prevalence was similar across regional studies compared with the corresponding national data (ATPIII 2001: 16.0% vs. 17.1%, p = 0.607; ATPIII 2004–2005: 24.3% vs. 25.4%, p = 0.861; IDF 2005: 17.4% vs. 18.7%, p = 0.785) (S6 Table). According to the criteria used, the prevalence of MS within a country differed in regional studies (p = 0.024) but was not significantly different in national studies (p = 0.109).

### Year of publication

The prevalence of MS in studies reported before, compared to after, 2010 was significantly higher by the ATPIII 2004–2005 criteria (30.6% vs. 21.5%, p = 0.012) but not by the other definitions (all p≥0.100). However, MS prevalence in the earlier publications differed significantly by criteria (16.4% vs. 30.6% vs. 14.5% for ATPIII-2001, ATPIII-2004-2005, IDF-2005 respectively, p<0.001).

### Sample size and smoking status

The median number of participants per study was 292 with this sample size used to classify studies as either large or small. MS prevalence was similar in large and small studies within criteria: ATPIII 2001:18.3% vs. 15.1%, p = 0.115; ATPIII 2004–2005: 24.7% vs. 25.6%, p = 0.989; IDF 2005: 19.0% vs. 16.1%, p = 0.388. There was no significant difference in prevalence by sample size category across MS criteria (all p≥0.059).

### Smoking status

The absence of a significant difference in the prevalence of MS in studies with a high or low proportion of smokers (median 39.8%) within criteria is demonstrated in S6 Table. The prevalence was as follows: ATPIII 2001: 14.8% vs. 17.6%, p = 0.234; ATPIII 2004–2005: 28.8% vs. 22.2%, p = 0.193; IDF 2005: 18.4% vs. 14.6%, p = 0.565. However, MS prevalence varied significantly across criteria in studies with a higher proportion of smokers (p = 0.010). There was substantial heterogeneity within all the above subgroups (all p-*heterogeneity*<0.001).

## Discussion

### Overview of MS prevalence

To our knowledge, this is the first comprehensive review and meta-analysis of the MS prevalence in the global HIV-infected population. Among the key findings was the high burden of MS in the HIV-infected population; 16.7–31.3% of HIV-infected adults had MS by the various definitions. The wide prevalence range is indicative of substantial heterogeneities across and within the diagnostic criteria. Two different criteria, the ATPIII-2001 and the EGIR-2003, reported the overall lowest and highest prevalences, respectively. Notably, the differences in prevalences were not fully explained by the major study characteristics. For example, variations in MS prevalence were also apparent within subgroups such as in younger participants, men, the Americas, regional studies, older publications and smokers.

Notably, the MS prevalence in the HIV-infected is within the range of the 17–46% reported in the general population. This highlights that the risk for developing MS in HIV-infected individuals is likely comparable to that in the general population. It also underscores the importance of traditional cardio-metabolic risk factors in the former; these are likely to exert an equal influence on HIV-infected individuals as they do in the general population. The wide range in MS prevalence in the general population mimics that found in this review and is possibly due to similar reasons discussed above [33–35].

The similar exposure to cardio-metabolic risk factors in the HIV-infected compared to the general population is likely attributable to the introduction of ART which has dramatically reduced HIV-related morbidity and mortality. ART has prolonged lifespans and subsequently enabled the HIV-infected to be exposed to the same risk factors and diseases as the general population. Reinforcing the likelihood of similar pathways in the development of MS in the HIV-infected and general populations was the higher MS prevalence in older compared to younger HIV-infected individuals, which is mirrored in general populations [33].

The higher MS prevalence in women compared to men in this analysis has also been shown in general populations but reports diverge in the latter [33–35]. Further research may be required to understand the differences in MS prevalence by gender which is usually driven by higher rates of obesity [36]. There may also be HIV specific factors that contribute to greater cardio-metabolic abnormalities in women compared to men that require further investigation.

### MS prevalence by diagnostic criteria

Across the three major criteria (ATPIII 2001, ATPIII 2004–2005, and IDF 2005) used by most studies in this review, the estimated MS prevalence was highest by the ATPIII 2004–2005 definition (24.6%). This was not unexpected because the threshold for hyperglycaemia in the ATPIII 2004–2005 is lower than that for the ATPIII 2001criteria; this leads to more individuals diagnosed with MS by the former classification. Furthermore, the inclusion of lipid-lowering and/or antihypertensive medications in the ATPIII 2004–2005 definition also contributes to a higher MS diagnosis compared with the ATPIII 2001. In contrast, the compulsory incorporation of waist circumference in the IDF 2005 criteria excludes many HIV-infected participants with abnormal biochemical parameters but normal waist circumference from the diagnosis. This is of particular relevance and frequently reported in HIV studies [25,37–41].

Not surprisingly, few studies that used more than one definition to diagnose MS applied the same combination of criteria [25,42]. In this review, direct comparison of MS prevalence by the various criteria is not meaningful because there was substantial overlap of the confidence intervals around the prevalence estimates. Also, only few studies used multiple diagnostic criteria which would limit the value of such an analysis.

### HIV-related influences on MS prevalence

Although HIV specific characteristics were associated with the prevalence of MS in the current analyses, these need to be interpreted with care because of the inability to control for the many confounding influences. Nonetheless, the association of MS prevalence with a greater duration of diagnosed HIV infection accords with the influence of HIV infection on the development of cardio-metabolic abnormalities. Then again, a longer interval since HIV diagnosis likely correlates with older age, which is a risk factor for MS in both the HIV-infected and general populations. It may also possibly reflect the specific effects of prolonged ART.

The relation between CD4 count and MS was unclear with some studies reporting a direct link [43,44] while others demonstrate the inverse [45] or no association [46,47]. However, these findings were based on only seven studies and did not report the viral loads [13,17,22,40,42,48,49]. Without such information it is difficult to draw conclusions on this relationship. A high viral load has been associated with the development of MS, possibly contributing to the high incidence of low high density lipoprotein cholesterol (HDL-C) levels and high triglycerides in some studies [16,50].

The higher MS prevalence in ART-exposed compared to ART-naïve participants by the ATPIII-2001 criteria was consistent with findings from prospective studies. One of these studies demonstrated an increase in MS prevalence from 16.6% to 25% with an incidence of 14/100 patients-year among participants initiated and maintained on the same HAART regimen for 48 weeks [51]. In another study, a large international, multicentre, randomised trial conducted for three years after the initiation of ART, the incidence of MS was 12/100 patients-year and 8/100 patients-year according to the ATPIII 2001 and the IDF 2005 criteria, respectively [28]. Also, the D:A:D study which followed HIV patients on ART over a long period, demonstrated a substantial increase in MS prevalence [52].

ART regimen was significantly associated with MS with notable differential findings by the class of drug used. The higher MS prevalence in studies with a greater compared to smaller proportion of PI users accords with four trials where PI-based regimens were found to accelerate progression to MS [37,38]. The initiation of ART leads to chronic inflammation and an incompletely restored immune system. This may perhaps be the link between PI use and the development of MS [53,54]. Furthermore, PI drugs have been reported to be associated with more severe dyslipidaemia compared to NNRTI, which is a feature of metabolic syndrome. This would explain at least in part the higher prevalence of MS in patients on PI. Patients on this regimen thus need to be closely monitored for the development of cardio-metabolic abnormalities. Moreover, once such abnormalities arise, it is important to review ART management and change to metabolically neutral regimens.

An alternative ART regimen to PIs in patients with MS may be NNRTIs because a lower MS prevalence, by some criteria, was detected in studies with a high compared to low proportion of participants on these drugs. Although our analyses were based on only seven studies [14,20,22,29,37,41,44] this suggests that, unlike PIs, NNRTIs do not adversely influence cardio-metabolic function and may possibly even have a beneficial impact. Indeed, a randomised controlled study reported improvements in cardio-metabolic profiles with increases in HDL-C levels in patients on nevirapine and nelfinavir [55]. On the other hand, several prospective trials have found no association between the use of NNRTIs and MS [37,38]. Further research is required to clearly describe the relationship between NNRTIs and cardio-metabolic functioning, particularly since there is a dearth of data on the influence of this class of ART on MS.

### Other influences on MS prevalence

Although there was no difference in MS prevalence between studies with a high and low proportion of smokers, conclusions on the absence of an association should be cautioned against. The studies analysed included only current smokers with no consideration given to recent smoking cessation or ex-smokers. Reports describe the duration of smoking cessation to be inversely related to future cardiovascular disease risk to a moderate degree. Furthermore, aspects not considered in this review such as the smoking interval and the quantity smoked have been strongly correlated with the development of MS and atherosclerosis progression [56–58].

The lower MS prevalence by the ATPIII 2004–2005 criteria in recent compared to older publications was surprising. We expected the trend to mimic that of the general population with a rise in MS prevalence in the HIV-infected over time. Moreover, with the introduction and widespread uptake of ART leading to longevity, we anticipated the subsequent development of MS with age, which would be reported in recent publications. Nevertheless, our findings are elucidated by the differences in participant characteristics between the two publication periods. Participants in publications prior to 2010 were older and included more women who were shown to have a higher MS prevalence than men in this review.

Despite an unbalanced representation of studies worldwide, the prevalence of MS was essentially similar within and across the major regions including by continent and intra-country site, regardless of the definition criteria used. The absence of studies conducted specifically in rural settings with a likely lower MS prevalence than in urban centres, particularly in developing regions, may account for this finding. Alternatively, it may perhaps reflect the ubiquitous worldwide influence of globalisation and highlight the likelihood of similar influences on the development of MS in the HIV-infected population globally. Thus, broad-based general strategies may perhaps be devised to address the MS burden in all HIV-infected populations.

### Strengths and limitations

#### Strengths

We searched multiple databases extensively, applying reproducible criteria to capture the most number of studies on MS prevalence worldwide. This allowed us to provide a comprehensive global perspective on the emerging burden of adverse cardio-metabolic profiles in the HIV-infected population. Furthermore, we used robust approaches to pool studies while minimising the effects of extreme studies. We also used a detailed approach to investigate the potential sources of heterogeneity. Our post-hoc power estimation revealed that over two-thirds of the included studies were adequately powered to provide precise estimates of the MS prevalence in the overall sample. This has likely translated into stable and robust pooled estimates by combining those primary studies.

#### Limitations

Our findings may not be generalizable to all HIV-infected individuals because most of the studies were conducted in non-randomly selected populations. The wide range in MS prevalence, because of the different criteria used across studies, although expected, made estimations of the actual burden difficult. Nevertheless, apart from differences in the criteria themselves that contributed to this wide range, other factors were also likely responsible. For example, MS prevalence would be expected to differ across time, particularly in the HIV-infected as access to care expanded, the uptake of ART increased and the effectiveness of therapy improved with the introduction of HAART. In some included studies, participants were selected with consideration to histories of existing CVD risk factors, which in turn can result in MS prevalence rates different from those in a broader population of HIV-infected individuals. This could possibly contribute to some of the heterogeneities observed across studies.

The infrequent reporting of the HIV specific markers of CD4 count and viral load precluded in-depth analyses of their effects on MS. Similarly, data were missing on key study characteristics which could be used in advanced analyses via meta-regressions. Furthermore, the inconsistent number of studies across subgroups precluded meta-regression analyses to investigate the possible contribution of each factor to MS prevalence. However, such comparisons would possibly have been biased by differences in study design and objectives, data collection techniques, laboratory facilities and participant characteristics, and could not have been fully accounted for in our meta-analyses. Especially difficult to control for would be HIV related characteristics such as differences in disease stage, fat distribution including lipodystrophy, obesity and co-existent infections such as hepatitis C and hepatitis B [59].

## Conclusion and Implications

The MS prevalence in HIV-infected individuals worldwide appears to be similar to that found in the general population, suggesting similarities in the drivers of the syndrome, independent of HIV status. Indeed, despite suggestions of significant signals, the inconsistent association of HIV specific features including treatments with MS prevalence suggest that their contribution, if any, is of a lesser magnitude. Comparable with general populations, traditional risk factors are likely the major contributors to the burden of cardio-metabolic abnormalities and MS in HIV-infected individuals. Therefore, management strategies implemented in the general population for these conditions, will likely reap similar benefits in the HIV-infected. Nevertheless, the major challenge lies in devising and strengthening approaches to maximise cardio-metabolic care while simultaneously ensuring optimal HIV management.

## Supporting Information

S1 Fig Overall prevalence of metabolic syndrome based on Joint Interim Statement (JIS) 2009 criteria. (PDF)

S2 Fig Overall prevalence of metabolic syndrome based on European Group for the Study of Insulin Resistance (EGIR) criteria. (PDF)

S3 Fig Overall prevalence of metabolic syndrome based on Modified Adult Treatment Panel III (ATPIII) 2005 criteria. (PDF)

S1 Table Details of the search strategies. (PDF)

S2 Table Quality assessment checklist for prevalence studies (adapted from Hoy et al [1]). (PDF)

S3 Table Different criteria for clinical diagnosis of the metabolic syndrome. (PDF)

S4 Table Summary of characteristics of the studies included in the present review. (PDF)

S5 Table The prevalence of metabolic syndrome estimated by the included studies according to criteria. (PDF)

S6 Table Summary statistics from meta-analyses of prevalence studies on metabolic syndrome in people with HIV using random effects model and arcsine transformations. (PDF)
