# Supplementary material for: A Meta-Analysis of the Metabolic Syndrome Prevalence in the Global HIV-Infected Population
Source: PLoS One. 2016 Mar 23;11(3):e0150970. doi: 10.1371/journal.pone.0150970 (PMC4805252; doi:10.1371/journal.pone.0150970)

S1 Fig. Overall prevalence of metabolic syndrome based on Joint Interim Statement (JIS) 2009 criteria

For each study the black box represents the study estimate (prevalence of metabolic syndrome [MS]) and the horizontal bar about the 95% confidence intervals. (95%CI) The size of the boxes is proportional to the inverse variance. The diamond at the lower tail of the figure is for the pooled effect estimates from random effects models. The proportional contribution of each study (weight) to the pooled estimates is also shown, together with the prevalence estimates and measures of heterogeneity. The dotted vertical line is centred on the pooled estimates.

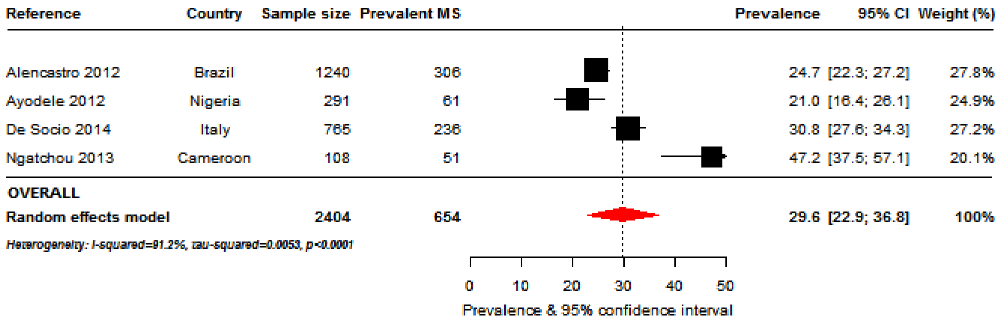

Supplement: S1 Fig — (PDF) [file pone.0150970.s001.pdf]
