# Supplementary material for: A Meta-Analysis of the Metabolic Syndrome Prevalence in the Global HIV-Infected Population
Source: PLoS One. 2016 Mar 23;11(3):e0150970. doi: 10.1371/journal.pone.0150970 (PMC4805252; doi:10.1371/journal.pone.0150970)
Supplement: S1 Table — (PDF) [file pone.0150970.s004.pdf]

S1 Table. Details of the search strategies

PubMed

Search (((((((("Metabolic Syndrome X"[Mesh]) OR Metabolic Syndrome) OR Reaven Syndrome) OR Cardiometabolic syndrome) OR cardiometabolic disease) OR syndrome X)) AND (((((HIV[MeSH Terms]) OR HIV) OR human immunodeficiency virus)) OR (((acquired immunodeficiency syndrome[MeSH Terms]) OR acquired immunodeficiency syndrome) OR AIDS))) AND ((((prevalence[MeSH Terms]) OR prevalence) OR epidemiology[MeSH Terms]) OR epidemiology) Filters: Publication date from 1998/01/01 to 2015/04/30

Scopus

(TITLE-ABS-KEY (prevalence OR epidemiology)) AND ((TITLE-ABS-KEY(hiv OR aids)) AND ((TITLE-ABS-KEY(metabolic syndrome x)) OR (TITLE-ABS-KEY(metabolic syndrome)))) AND (LIMIT-TO(PUBYEAR, 2015) (LIMIT-TO(PUBYEAR, 2014) OR LIMIT-TO(PUBYEAR, 2013) OR LIMIT-TO(PUBYEAR, 2012) OR LIMIT-TO(PUBYEAR, 2011) OR LIMIT-TO(PUBYEAR, 2010) OR LIMIT-TO(PUBYEAR, 2009) OR LIMIT-TO(PUBYEAR, 2008) OR LIMIT-TO(PUBYEAR, 2007) OR LIMIT-TO(PUBYEAR, 2006) OR LIMIT-TO(PUBYEAR, 2005) OR LIMIT-TO(PUBYEAR, 2014) OR LIMIT-TO(PUBYEAR, 2013) OR LIMIT-TO(PUBYEAR, 2012) OR LIMIT-TO(PUBYEAR, 2011) OR LIMIT-TO(PUBYEAR, 2010) OR LIMIT-TO(PUBYEAR, 2009) OR LIMIT-TO(PUBYEAR, 2008) OR LIMIT-TO(PUBYEAR, 2007) OR LIMIT-TO(PUBYEAR, 2006) OR LIMIT-TO(PUBYEAR, 2005) OR LIMIT-TO(PUBYEAR, 2004) OR LIMIT-TO(PUBYEAR, 2003) OR LIMIT-TO(PUBYEAR, 2002) OR LIMIT-TO(PUBYEAR, 2001) OR LIMIT-TO(PUBYEAR, 2000) OR LIMIT-TO(PUBYEAR, 1999) OR LIMIT-TO(PUBYEAR, 1998) OR LIMIT-TO(PUBYEAR, 2014) OR LIMIT-TO(PUBYEAR, 2013) OR LIMIT-TO(PUBYEAR, 2012) OR LIMIT-TO(PUBYEAR, 2011) OR LIMIT-TO(PUBYEAR, 2010) OR LIMIT-TO(PUBYEAR, 2009) OR LIMIT-TO(PUBYEAR, 2008) OR LIMIT-TO(PUBYEAR, 2007) OR LIMIT-TO(PUBYEAR, 2006) OR LIMIT-TO(PUBYEAR, 2005) OR LIMIT-TO(PUBYEAR, 2004) OR LIMIT-TO(PUBYEAR, 2003) OR LIMIT-TO(PUBYEAR, 2002) OR LIMIT-TO(PUBYEAR, 2001) OR LIMIT-TO(PUBYEAR, 2000) OR LIMIT-TO(PUBYEAR, 1999) OR LIMIT-TO(PUBYEAR, 1998))

EbscoHost

S9 S3 AND S8 (prevalence OR epidemiology) AND (((HIV OR human immunodeficiency virus) OR (AIDS OR acquired immunodeficiency syndrome)) AND (Metabolic syndrome X OR metabolic Syndrome))

S8 S6 AND S7 (((HIV OR human immunodeficiency virus) OR (AIDS OR acquired immunodeficiency syndrome)) AND (Metabolic syndrome X OR metabolic Syndrome))

S7 S2 OR metabolic syndrome (Metabolic syndrome X OR metabolic Syndrome)

S6 S4 OR S5 ((HIV OR human immunodeficiency virus) OR (AIDS OR acquired immunodeficiency syndrome))

S5 AIDS OR acquired immunodeficiency syndrome

S4 HIV OR human immunodeficiency virus

S3 prevalence OR epidemiology

S2 (MM "Metabolic Syndrome X+")
