# Supplementary material for: A Meta-Analysis of the Metabolic Syndrome Prevalence in the Global HIV-Infected Population
Source: PLoS One. 2016 Mar 23;11(3):e0150970. doi: 10.1371/journal.pone.0150970 (PMC4805252; doi:10.1371/journal.pone.0150970)
Supplement: S3 Table — (PDF) [file pone.0150970.s006.pdf]

S3 Table. Different criteria for clinical diagnosis of the metabolic syndrome

Clinical measure	WHO (1998) [1]	EGIR (1999) [2]	ATP III (2001) [3]	AACE (2003) [4]	Modified ATP III (2004) [5]	IDF (2005) [6]	NCEP-ATPIII (2005) [7]	JIS (2009) [8]
1. Insulin resistance	IGT, IFG, T2DM, or low insulin sensitivity [a] plus any 2 of the following:	Plasma insulin >75th percentile plus any 2 of following features	None, but any 3 of the following:	IGT or IFG plus any of the following based on clinical assessment:	None, but any 3 of 5 the following:	None	None, but any 3 of the following:	None, but any 3 of the following:
2. Body composition	WHR >0.90 in men, >0.85 in women and/or BMI >30 kg/m^2	WC ≥94 cm in men, ≥80 cm in women	WC ≥102 cm in men, ≥88 cm in women	BMI ≥25 kg/m^2	WC ≥102 cm in men, ≥88 cm in women	Increased WC >94 cm in men, ≥80 cm in women plus any 2 of the following:	WC ≥102 cm in men, ≥88 cm in women	Population-and country-specific definitions[c]
3. Lipids	TG ≥ 1.7 mmol/L and/or HDL-C <0.9 mmol/L in men, <1 mmol/L in women	TG ≥2.0 mmol/L and/or HDL-C <1.01 mmol/L or treated for dyslipidemia	TG ≥1.69 mmol/L, HDL-C <1.03 mmol/L in men, <1.3 mmol/L in women	TG ≥1.7 mmol/L and HDL-C <1 mmol/L	TG ≥1.69 mmol/L; HDL-C <1.03 mmol/L in men, <1.3 mmol/L in women	TG ≥1.7 mmol/L or TG Rx; HDL-C <1.03 mmol/L in men, <1.3 mmol/L in women or HDL-C Rx	TG ≥1.7 mmol/L or on TG Rx; HDL-C <1 mmol/L in men, <1.3 mmol/L in women or HDL-C Rx	TG ≥ 1.7 mmol/L or on TG Rx; HDL-C < 1.03 mmol/L in men, <1.3 mmol/L in women or HDL-C Rx
4. Blood pressure	SBP/DBP≥160/90 mmHg	SBP/DBP≥140/90 mmHg or hypertension Rx	SBP/DBP ≥130/85 mmHg	SBP/DBP ≥130/85 mmHg	SBP/DBP≥130/85 mmHg	SBP/DBP ≥130/85 mmHg or hypertension Rx	SBP/DBP ≥130/85 mmHg or hypertension Rx	SBP/DBP ≥130/85 mmHg or hypertension Rx
5. Glucose metabolism	IGT, IFG, or T2DM	IGT or IFG (but not diabetes)	>6.11 mmol/L (includes diabetes)	IGT or IFG (but not diabetes)	>5.6 mmol/L (includes diabetes)	≥5.6 mmol/L (includes diabetes)	≥ 5.6 mmol/L or on hyperglycemic Rx	≥ 5.6 mmol/L or on hyperglycemic Rx
6. Other	mALB			Other features of Ins-R[b]				

AACE; American Association of Clinical Endocrinologists; ATP III, Adults Treatment Panel III; BMI, body mass index; DBP, diastolic blood pressure; EGIR, European Group for the Study of Insulin Resistance; HDL-C, high density lipoprotein cholesterol; IDF, International Diabetes Federation; IGT indicates impaired glucose tolerance; IFG, impaired fasting glucose; Ins-R, Insulin resistance; JIS, Joint Interim Statement; NCEP-ATPIII, National Cholesterol Education Program, Adults Treatment Panel III; mALB, micro albumin; Rx, prescription; SBP, systolic blood pressure; TG, triglycerides; T2DM indicates type 2 diabetes mellitus; WC, waist circumference; WHR, waist-to-hip ratio; WHO, World Health Organisation

a Insulin sensitivity measured under hyperinsulinaemic euglycaemic conditions; glucose uptake below lowest quartile for background population under investigation.

b Includes family history of T2DM mellitus, sedentary lifestyle, advancing age, and ethnic groups susceptible to T2DM

[C] European, Middle East, Mediterranean, sub-Saharan African: WC ≥94 cm in men, ≥80 cm in women; Asian, Ethnic Central and South America: WC ≥90 cm in men, ≥80 cm in women

References

1. Alberti KG, Zimmet PZ. Definition, diagnosis and classification of diabetes mellitus and its complications. Part 1: diagnosis and classification of diabetes mellitus provisional report of a WHO consultation. Diabet Med. 1998;15: 539-553.
2. Balkau B, Charles MA. Comment on the provisional report from the WHO consultation. European Group for the Study of Insulin Resistance (EGIR). Diabetic Medicine: A Journal Of The British Diabetic Association. 1999;16: 442-443.
3. Third Report of the National Cholesterol Education Program (NCEP) Expert Panel on Detection, Evaluation, and Treatment of High Blood Cholesterol in Adults (Adult Treatment Panel III) final report. Circulation. 2002;106: 3143-3421.
4. Einhorn D, Reaven GM, Cobin RH, Ford E, Ganda OP, Handelsman Y, et al. American College of Endocrinology position statement on the insulin resistance syndrome. Endocrine Practice: Official Journal Of The American College Of Endocrinology And The American Association Of Clinical Endocrinologists. 2003;9: 237-252.
5. Grundy SM, Hansen B, Smith SC, Jr., Cleeman JI, Kahn RA. Clinical management of metabolic syndrome: report of the American Heart Association/National Heart, Lung, and Blood Institute/American Diabetes Association conference on scientific issues related to management. Circulation. 2004;109: 551-556.
6. Alberti KG, Zimmet P, Shaw J. The metabolic syndrome--a new worldwide definition. Lancet (London, England). 2005;366: 1059-1062.
7. Grundy SM, Cleeman JI, Daniels SR, Donato KA, Eckel RH, Franklin BA, et al. Diagnosis and management of the metabolic syndrome: an American Heart Association/National Heart, Lung, and Blood Institute Scientific Statement. Circulation. 2005;112: 2735-2752.
8. Alberti KGMM, Eckel RH, Grundy SM, Zimmet PZ, Cleeman JI, Donato KA, et al. Harmonizing the metabolic syndrome: a joint interim statement of the International Diabetes Federation Task Force on Epidemiology and Prevention; National Heart, Lung, and Blood Institute; American Heart Association; World Heart Federation; International Atherosclerosis Society; and International Association for the Study of Obesity. Circulation. 2009;120: 1640-1645.
