# Supplementary material for: A Meta-Analysis of the Metabolic Syndrome Prevalence in the Global HIV-Infected Population
Source: PLoS One. 2016 Mar 23;11(3):e0150970. doi: 10.1371/journal.pone.0150970 (PMC4805252; doi:10.1371/journal.pone.0150970)
Supplement: S4 Table — (PDF) [file pone.0150970.s007.pdf]

S4 Table. Summary of characteristics of the studies included in the present review

Reference	Substudies/ subgroups	Sample size	Male (%)	Age (years)	Current smoker	Lipodystrophy	HIV/HCV	CD4 count (cells/µL)	Viral load (copies/mm^3)	HIV period (months)	ART Period (months)	ART use (%)	PIs use (%)	NNRTIs use (%)	NRTIs use (%)	HIV disease stage				
																Stage 1+2	Stage 3+4	Stage A	Stage B	Stage C
			%	mean	%	%	%	mean	mean (Log10)	mean	mean	%	%	%	%	%	%	%	%	%
Adeyemi, et al, 2008 [1]	Overall	121	79	54	65	NR	NR	382	NR	144	NR	NR	NR	NR	NR	NR	NR	NR	NR	NR
	Men	95	NA	54	66	NR	NR	383	NR	140	NR	NR	NR	NR	NR	NR	NR	NR	NR	NR
	Women	26	NA	54	61	NR	NR	376	NR	153.6	NR	NR	NR	NR	NR	NR	NR	NR	NR	NR
Alencastro, et al, 2012 [2]	Overall	1240	51	38.6	NR	NR	NR	NR	NR	58.8	NR	65.7	37.7	NR	NR	NR	NR	NR	NR	NR
	Men	628	NA	39.5	NR	NR	NR	NR	NR	62.4	NR	66.9	33.8	NR	NR	NR	NR	NR	NR	NR
	Women	612	NA	37.7	NR	NR	NR	NR	NR	55.2	NR	64.5	42.0	NR	NR	NR	NR	NR	NR	NR
Ances, et al, 2009 [3]	Overall	66	82	41	NR	NR	NR	NR	NR	NR	NR	NR	NR	NR	NR	NR	NR	NR	NR	NR
Awotedu, et al, 2010 [4]	Overall	196	19	36.8	NR	NR	NR	366	NR	NR	NR	NR	NR	NR	NR	NR	NR	NR	NR	NR
	ART	86	13	37.7	NR	NR	NR	350	NR	NR	NR	NR	NR	NR	NR	NR	NR	NR	NR	NR
	No-ART	110	24	36	NR	NR	NR	379	NR	NR	0	0	NR	NR	NR	NR	NR	NR	NR	NR
Ayodele, et al, 2012 [5]	Overall	291	33	39.5	1.4	NR	NR	326	NR	NR	17.2	81	NR	NR	NR	NR	NR	NR	NR	NR
	Men	95	NA	42.5	4.3	NR	NR	250	NR	NR	15.6	81	NR	NR	NR	NR	NR	NR	NR	NR
	Women	196	NA	38	0.5	NR	NR	362	NR	NR	18	81	NR	NR	NR	NR	NR	NR	NR	NR
Badiou, et al, 2008 [6]	Overall	232	75	41	70	37	NR	465	2.9	NR	37	80	46	31	NR	NR	NR	NR	NR	NR
Bajaj, et al, 2013 [7]	Overall	70	71.4	NR	NR	NR	NR	NR	NR	NR	NR	NR	NR	NR	NR	NR	NR	NR	NR	NR
Baum, et al, 2006 [8]	Overall	118	74	41.7	84	NR	NR	333	9.3	NR	NR	71	36.5	NR	NR	NR	NR	NR	NR	NR
	Men	87	100	NR	NR	NR	NR	316	9.2	NR	NR	NR	NR	NR	NR	NR	NR	NR	NR	NR
	Women	31	0	NR	NR	NR	NR	382	9.6	NR	NR	NR	NR	NR	NR	NR	NR	NR	NR	NR

Bergersen,et al, 2006 [9]	Overall	263	81	43.3	54.7	NR	NR	380	2.6	61.4	26	78.7	NR	NR	NR	NR	NR	NR	NR	NR
	ART	207	81.6	43.4	55.1	NR	NR	380	2.1	85.5	33	100	77	39	NR	NR	NR	NR	27.5	NR
	No-ART	56	76.8	42.8	53.6	NR	NR	380	4.6	37.3	0	0	NA	NA	NR	NR	NR	NR	1.8	NR
Berhane, et al, 2012 [10]	Overall	313	34.8	NR	NR	12.1	NR	NR	NR	NR	NR	NR	NR	NR	NR	43.5	56.5	NA	NA	NA
Biron, et al, 2012 [11]	Overall	269	66.9	43	48.3	14.5	NR	438	2.4	56.4	29.8	100	NR	NR	NR	NA	NA	51.3	24.2	24.5
	ART	269	66.9	43	48.3	14.5	NR	438	2.4	56.4	29.8	100	NR	NR	NR	NA	NA	51.3	24.2	24.5
Bonfanti, et al, 2010 [12]	Overall	292	75	37	50.7	NR	NR	NR	NR	NR	NA	NA	NA	NA	NA	NA	NA	80	10.6	9.4
Bonfanti, et al, 2007 [13]	Overall	1243	71.8	43.2	60.2	NR	NR	NR	NR	NR	NR	NR	NR	NR	NR	NR	NR	NR	NR	NR
Cahn, et al, 2010 [14]	Argentina	1015	70.4	41.6	34.1	NR	NR	447	2.1	NR	23	100	36.2	61.9	NR	NR	NR	NR	NR	NR
	Brazil	1001	65.4	44	22.1	NR	NR	474	2.3	NR	40	100	51.4	51.8	NR	NR	NR	NR	NR	NR
	Colombia	474	86.7	40.8	18.9	NR	NR	390	2.2	NR	23	100	38	58.9	NR	NR	NR	NR	NR	NR
	Chile	44	90.9	40.6	50.0	NR	NR	362	2.2	NR	24.5	100	20	81.8	NR	NR	NR	NR	NR	NR
	Ecuador	252	77.8	39	10.3	NR	NR	363	2.7	NR	19	100	30.6	70.6	NR	NR	NR	NR	NR	NR
	Peru	417	70.7	39.1	15.6	NR	NR	255	2.8	NR	18	100	31.9	71.7	NR	NR	NR	NR	NR	NR
	Venezuela	807	80.9	42.6	18.2	NR	NR	452	2.4	NR	27	100	61.5	38.2	NR	NR	NR	NR	NR	NR
Calza, et al, 2011 [15]	Overall	755	66.2	37	35	40.5	NR	453	4.1	85.3	66	87	41.2	43.4	NR	NA	NA	68	28	4.0
Cubero, et al, 2011 [16]	Overall	159	75.5	39	64.2	44.6	NR	388	3.7	63	NR	100	NR	NR	NR	NR	NR	NR	NR	NR
	ART	159	75.5	39	64.2	44.6	NR	388	3.7	63	NR	100	NR	NR	NR	NR	NR	NR	NR	NR
Da Silva, et al, 2009 [17]	Overall	319	60.9	39.5	27	NR	NR	502.9	NR	61	NR	76.2	NR	NR	NR	NR	NR	NR	NR	NR
	ART	243	59.7	41	25.5	NR	NR	476.5	NR	69.6	NR	100	NR	NR	NR	NR	NR	NR	NR	NR
	No-ART	76	65.8	34.8	30.3	NR	NR	587.2	NR	33.6	0	0	NR	NR	NR	NR	NR	NR	NR	NR
De Socio, et al, 2014 [18]	Overall	765	74	45.6	49.9	30.8	NR	NR	NR	NR	NR	94	53.4	38.8	1.5	NR	NR	NR	NR	NR
Elgalib, et al, 2011 [19]	Overall	678	74	39.5	38.3	NR	NR	NR	NR	NR	26	74	37	38	NR	NR	NR	NR	NR	NR
Estrada, et al, 2006 [20]	Overall	146	65.8	40.6	67.1	67.3	NR	527	2.13	NR	NR	100	NR	NR	NR	NR	NR	NR	NR	NR

	ART	146	65.8	40.6	67.1	67.3	NR	527	2.13	NR	NR	100	NR	NR	NR	NR	NR	NR	NR	NR
Fourie, et al, 2010 [21]	Overall	300	38.7	44	42.3	NR	NR	NR	NR	NR	NA	NA	NA	NA	NA	NR	NR	NR	NR	NR
Freitas, et al, 2011 [22]	Overall	345	69.3	43.8	43.2	58.7	NR	NR	NR	NR	NR	NR	NR	NR	NR	NR	NR	NR	NR	NR
Gasparotto, et al, 2012 [23]	Overall	614	55.5	42.6	NR	NR	NR	NR	NA	NR	NR	100	NR	NR	NR	NR	NR	NR	NR	NR
	ART	614	55.5	42.6	NR	NR	NR	NR	NA	NR	NR	100	NR	NR	NR	NR	NR	NR	NR	NR
	Men	341	100	43.6	NR	48.5	NR	NR	NR	NR	59	100	47.5	NR	NR	NR	NR	NR	NR	NR
	Women	237	0	41.3	NR	54.8	NR	NR	NR	NR	59.5	100	51.6	NR	NR	NR	NR	NR	NR	NR
Gazzaruso, et al, 2003 [24]	Overall	287	70.7	41	NR	41.1	NR	477	3.97	NR	NR	100	NR	NR	NR	NR	NR	NR	NR	NR
	ART	287	70.7	41	NR	41.1	NR	477	3.97	NR	NR	100	NR	NR	NR	NR	NR	NR	NR	NR
Guaraldi, et al, 2012 [25]	Overall	133	100	NR	39.8	85	NR	NR	NR	NR	NR	NR	NR	NR	NR	NR	NR	NR	NR	NR
Guaraldi, et al, 2011 [26]	Overall	143	66.4	NR	NR	NR	28	NR	NR	NR	NR	100	NR	NR	NR	NR	NR	NR	NR	NR
	ART	143	66.4	NR	NR	NR	28	NR	NR	NR	NR	100	NR	NR	NR	NR	NR	NR	NR	NR
	HIV alone	103	68	46.9	NR	NR	NA	NR	2.13	NR	NR	100	NR	NR	NR	NR	NR	NR	NR	NR
	HIV/HCV	40	63	46.2	NR	NR	NA	NR	1.69	NR	NR	100	NR	NR	NR	NR	NR	NR	NR	NR
Gupta, et al, 2011 [27]	Overall	68	84	35.9	NR	NR	NR	109	NR	NR	NR	100	NR	NR	NR	NR	NR	NR	NR	NR
	ART	68	84	35.9	NR	NR	NR	109	NR	NR	NR	100	NR	NR	NR	NR	NR	NR	NR	NR
Hadigan, et al, 2013 [28]	Overall	182	64.3	45	28.6	NR	7.7	513	2.1	168	NR	NR	NR	NR	NR	NR	NR	NR	NR	NR
Hansen, et al, 2009 [29]	Overall	566	81.4	44.1	NR	NR	NR	494	NR	114	62.4	83	31.4	53.5	55.4	NR	NR	NR	NR	20.3
Jacobson, et al, 2006 [30]	Overall	477	72.5	NR	NR	NR	NR	NR	NR	NR	NR	72	NR	NR	NR	NR	NR	NR	NR	40
	ART	342	72.8	42	NR	NR	NR	NR	NR	NR	NR	100	52	27	7	NR	NR	NR	NR	41
	No-ART	135	71.9	44	NR	NR	NR	NR	NR	NR	0	0	0	0	0	NR	N	NR	NR	38
Janiszewski, et al, 2011 [31]	Overall	2322	63.8	NR	30.6	34.9	NR	NR	NR	NR	NR	100	NR	NR	NR	NR	NR	NR	NR	NR
	ART	2322	63.8	NR	30.6	34.9	NR	NR	NR	NR	NR	100	NR	NR	NR	NR	NR	NR	NR	NR
	Men	1481	100	NR	32.2	31.5	NR	NR	NR	NR	NR	100	NR	NR	NR	NR	NR	NR	NR	NR

	Women	841	0	NR	27.7	41	NR	NR	NR	NR	NR	100	NR	NR	NR	NR	NR	NR	NR	NR
Jantarapakde, et al, 2014 [32]	Overall	580	46.2	37	16.4	45.2	NR	394	NR	60	NR	70.7	NR	NR	NR	NA	NA	50	19	30.5
	ART	410	51	39	16.3	56.1	NR	394	1.6	60	NR	100	14.9	79	NR	NA	NA	37.1	22.4	40.2
	No-ART	170	34.7	34	16.5	18.8	NR	392	4.1	36	0	0	0	0	0	NA	NA	81.2	10.6	7.1
Jerico, et al, 2005 [33]	Overall	710	72	41.9	67.5	36.6	NR	479	NR	113	78	88.2	28.6	NR	NR	NA	NA	47.7	19.6	32.7
Johnsen, et al, 2006 [34]	Overall	97	0	41	47	NR	NR	390	2.7	96	NR	81	45	51	81	NR	NR	NR	NR	NR
Krishnan, et al, 2012 [35]	Overall	2247	82	NR	59	NR	NR	NR	NR	NR	0	0	0	0	0	NR	NR	NR	NR	NR
Lauda, et al, 2011 [36]	Overall	249	52.2	NR	NR	NR	NR	NR	NR	NR	NR	87	NR	NR	NR	NR	NR	NR	NR	NR
Leite, et al, 2010 [37]	Overall	100	63	NR	23	NR	NR	525	NR	72	50.2	77	30	NR	NR	NR	NR	NR	NR	NR
Maloberti, et al, 2013 [38]	Overall	108	82.4	NR	55.6	NR	NR	NR	NR	NR	NA	66.7	NR	NR	NR	NR	NR	NR	NR	NR
	ART	72	83	46.5	58.3	NR	NR	450	5.15	NR	77	100	65.2	31.9	97.2	NR	NR	NR	NR	NR
	No-ART	36	81	40.7	50	NR	NR	478	4.8	NR	0	0	0	0	0	NR	NR	NR	NR	NR
Martin, et al, 2008 [39]	Overall	140	72	NA	NA	39.3	NR	NA	NA	NA	NA	NA	NA	NA	NA	NR	NR	NR	NR	NR
Mbunkah, et al, 2014 [40]	Overall	173	29	38.7	2.9	NR	NR	358	NR	34.1	23.7	65	NR	NR	NR	100	NA	NA	NA	NA
Mondy, et al, 2007 [41]	Overall	471	64.5	40.2	42.5	NR	NR	NR	NR	NR	NR	73	35	41	NR	NR	NR	NR	NR	NR
Muhammad, et al, 2013 [42]	Overall	200	47	32.5	9	NR	NR	318	NR	NR	NR	50	NR	NR	NR	NR	NR	NR	NR	NR
	ART	100	46	32.8	10	NR	NR	376	NR	NR	NR	100	NR	NR	NR	NR	NR	NR	NR	NR
	No-ART	100	48	32.4	8	NR	NR	261	NR	NR	NR	0	NR	NR	NR	NR	NR	NR	NR	NR
Palacios, et al, 2007 [43]	Overall	60	83.3	40.9	78.3	NR	26.7	186	5.64	38.4	0	0	0	0	NR	NR	NR	NR	NR	43.3
Ngatchou, et al, 2013 [44]	Overall	108	26	39	0	NR	NR	353	NR	19.3	0	0	0	0	0	NR	NR	NR	NR	NR
Pullinger, et al, 2010 [45]	Overall	296	73.3	45.3	NR	NR	33.8	455	NR	145	NR	70.9	42.2	19.3	5.1	NR	NR	NR	NR	53.7
Ramirez-Marrero, et al, 2010 [46]	Overall	897	64	44.7	50	NR	21	473	NR	NR	NR	45	3	34	NR	NR	NR	NR	NR	NR

	Men	574	100	44.4	53	NR	23	467	NR	NR	NR	46	3	32	NR	NR	NR	NR	NR	NR
	Women	323	0	45.2	44	NR	17	486	NR	NR	NR	42	2	38	NR	NR	NR	NR	NR	NR
Samaras, et al, 2007 [47]	Overall	788	84	NR	30	57.2	NR	NR	NR	NR	NR	92.9	50.3	37.9	85.5	NR	NR	50.4	23.4	26.4
Sawadogo, et al, 2014 [48]	Overall	400	29	41.4	4.8	NR	NR	503	NR	NR	50.7	100	17.3	95.0	NR	51	49	NA	NA	NA
	ART	400	29	41.4	4.8	NR	NR	503	NR	NR	50.7	100	17.3	95.0	NR	51	49	NA	NA	NA
	Men	116	100	41.6	15.5	NR	NR	485	NR	NR	53.5	100	17.2	96.6	NR	50	50	NA	NA	NA
	Women	284	0	39.6	0.4	NR	NR	457	NR	NR	49.6	100	17.3	94.4	NR	51.4	48.6	NA	NA	NA
Schillaci, et al, 2008 [49]	Overall	39	67	37	49	NR	NR	NR	4.71	60	0	0	0	0	0	NR	NR	NR	NR	NR
	No-ART	39	67	37	49	NR	NR	NR	4.71	60	0	0	0	0	0	NR	NR	NR	NR	NR
Signorini, et al, 2012 [50]	Overall	819	54.6	41	26	38.5	NR	394	NR	NR	54	76.1	27.6	48.5	NR	NR	NR	NR	NR	NR
Sobieszczyk, et al, 2008 [51]	Overall	1725	0	40	47	NR	NR	474	3.1	NR	NR	48	24	NR	NR	NR	NR	NR	NR	NR
	Women	1725	100	40	47	NR	NR	474	3.1	NR	NR	48	24	NR	NR	NR	NR	NR	NR	NR
Sterling, et al, 2008 [52]	Overall	222	74	45.4	NR	NR	100	535	NR	224.4	NR	83	47	30	82	NR	NR	NR	NR	NR
Tesfaye, et al, 2014 [53]	Overall	374	33.7	32.6	2.7	NR	NR	NR	NR	NR	42.6	50.3	NR	NR	NR	NR	NR	NR	NR	NR
	ART	188	36.2	32.7	2.7	NR	NR	441.6	NR	NR	42.6	100	NR	NR	NR	NR	NR	NR	NR	NR
	No-ART	186	31.2	32.6	2.7	NR	NR	493	NR	NR	0	0	NR	NR	NR	NR	NR	NR	NR	NR
Tiozzo, et al, 2015 [54]	Overall	89	47	48	NR	NR	NR	NR	NR	204	NR	NR	NR	NR	NR	NR	NR	NR	NR	NR
Wand, et al, 2007 [55]	Overall	881	79	38.7	40.2	NR	NR	NR	NR	NR	NR	100	NR	NR	NR	NA	NA	62.4	20	20.8
	ART	881	79	38.7	40.2	NR	NR	NR	NR	NR	NR	100	NR	NR	NR	NA	NA	62.4	20	20.8
Worm, et al, 2010 [56]	Overall	33347	74	38	33.9	NR	NR	NR	NR	NR	NR	NR	58	32.2	72.9	NA	NA	NA	NA	24.6
Wu, et al, 2012 [57]	Overall	803	95	NR	33.5	NR	NR	NR	NR	NR	NR	80.7	57.8	51.1	81.7	NR	NR	NR	NR	NR
Young, et al, 2009 [58]	Overall	1644	NR	NR	NR	NR	NR	NR	NR	NR	NR	NR	43	38	NR	NR	NR	NR	NR	NR
Zannou, et al, 2009 [59]	Overall	79	40.5	38	8.9	NR	NR	105.3	NR	NR	14.6	100	NR	NR	NR	51.9	48.1	NA	NA	NA
	ART	79	40.5	38	8.9	NR	NR	105.3	NR	NR	14.6	100	NR	NR	NR	51.9	48.1	NA	NA	NA

ART, antiretroviral; HCV, hepatitis C virus; NA not applicable; NNRTI, non-nucleoside reserve transcriptase inhibitor; NR, not reported; NRTI, nucleoside reverse transcriptase inhibitors; PI, protease inhibitor.

References

1. Adeyemi O, Rezai K, Bahk M, Badri S, Thomas-Gossain N. Metabolic syndrome in older HIV-infected patients: data from the CORE50 cohort. AIDS Patient Care and STDs. 2008;22: 941-945.
2. Alencastro PR, Wolff FH, Oliveira RR, Ikeda ML, Barcellos NT, Brandao AB, et al. Metabolic syndrome and population attributable risk among HIV/AIDS patients: comparison between NCEP-ATPIII, IDF and AHA/NHLBI definitions. AIDS research and therapy. 2012;9: 29-6405-6409-6429.
3. Ances BM, Bhatt A, Vaida F, Rosario D, Alexander T, Marquie-Beck J, et al. Role of metabolic syndrome components in human immunodeficiency virus-associated stroke. Journal of neurovirology. 2009;15: 249-256.
4. Awotedu K, Ekpebegh C, Longo-Mbenza B, Iputo J. Prevalence of metabolic syndrome assessed by IDF and NCEP ATP 111 criteria and determinants of insulin resistance among HIV patients in the Eastern Cape Province of South Africa. Diabetes and Metabolic Syndrome: Clinical Research and Reviews. 2010;4: 210-214.
5. Ayodele OE, Akinboro AO, Akinyemi SO, Adepeju AA, Akinremi OA, Alao CA, et al. Prevalence and clinical correlates of metabolic syndrome in Nigerians living with human immunodeficiency virus/acquired immunodeficiency syndrome. Metabolic syndrome and related disorders. 2012;10: 373-379.
6. Badiou S, Thiebaut R, Aurillac-Lavignolle V, Dabis F, Laporte F, Cristol JP, et al. Association of non-HDL cholesterol with subclinical atherosclerosis in HIV-positive patients. The Journal of infection. 2008;57: 47-54.
7. Bajaj S, Tyagi SK, Bhargava A. Metabolic syndrome in human immunodeficiency virus positive patients. Indian journal of endocrinology and metabolism. 2013;17: 117-120.
8. Baum MK, Rafie C, Lai S, Xue L, Sales S, Page JB, et al. Coronary Heart Disease (CHD) Risk Factors and Metabolic Syndrome in HIV-Positive Drug Users in Miami. American journal of infectious diseases. 2006;2: 173-179.

9. Bergersen BM, Schumacher A, Sandvik L, Bruun JN, Birkeland K. Important differences in components of the metabolic syndrome between HIV-patients with and without highly active antiretroviral therapy and healthy controls. Scandinavian Journal of Infectious Diseases. 2006;38: 682-689.
10. Berhane T, Yami A, Alemseged F, Yemane T, Hamza L, Kassim M, et al. Prevalence of lipodystrophy and metabolic syndrome among HIV positive individuals on highly active anti-retroviral treatment in Jimma, south west Ethiopia. Pan African Medical Journal. 2012;13.
11. Biron A, Bobin-Dubigeon C, Volteau C, Piroth L, Perre P, Leport C, et al. Metabolic syndrome in French HIV-infected patients: prevalence and predictive factors after 3 years of antiretroviral therapy. AIDS Research and Human Retroviruses. 2012;28: 1672-1678.
12. Bonfanti P, De Socio GL, Marconi P, Franzetti M, Martinelli C, Vichi F, et al. Is metabolic syndrome associated to HIV infection per se? Results from the HERMES study. Current HIV research. 2010;8: 165-171.
13. Bonfanti P, Giannattasio C, Ricci E, Facchetti R, Rosella E, Franzetti M, et al. HIV and metabolic syndrome: a comparison with the general population. Journal of acquired immune deficiency syndromes (1999). 2007;45: 426-431.
14. Cahn P, Leite O, Rosales A, Cabello R, Alvarez CA, Seas C, et al. Metabolic profile and cardiovascular risk factors among Latin American HIV-infected patients receiving HAART. The Brazilian journal of infectious diseases : an official publication of the Brazilian Society of Infectious Diseases. 2010;14: 158-166.
15. Calza L, Masetti G, Piergentili B, Trapani F, Cascavilla A, Manfredi R, et al. Prevalence of diabetes mellitus, hyperinsulinaemia and metabolic syndrome among 755 adult patients with HIV-1 infection. International Journal of STD and AIDS. 2011;22: 43-45.
16. Cubero JM, Domingo P, Sambeat M, Ordoñez-Llanos J, Rodriguez-Espinosa J, Sánchez-Quesada JL, et al. Prevalence of metabolic syndrome among human immunodeficiency virus-infected subjects is widely influenced by the diagnostic criteria. Metabolic Syndrome and Related Disorders. 2011;9: 345-351.
17. Da Silva ÉFR, Bassichetto KC, Lewi DS. Lipid profile, cardiovascular risk factors and metabolic syndrome in a group of AIDS patients. Arquivos Brasileiros de Cardiologia. 2009;93: 113-118.
18. De Socio GV, Ricci E, Maggi P, Parruti G, Pucci G, Di Biagio A, et al. Prevalence, awareness, treatment, and control rate of hypertension in HIV-infected patients: The HIV-HY study. American Journal of Hypertension. 2014;27: 222-228.
19. Elgalib A, Aboud M, Kulasegaram R, Dimian C, Duncan A, Wierzbicki AS, et al. The assessment of metabolic syndrome in UK patients with HIV using two different definitions: CREATE 2 study. Current medical research and opinion. 2011;27: 63-69.

20. Estrada V, Martinez-Larrad MT, Gonzalez-Sanchez JL, de Villar NG, Zabena C, Fernandez C, et al. Lipodystrophy and metabolic syndrome in HIV-infected patients treated with antiretroviral therapy. Metabolism: clinical and experimental. 2006;55: 940-945.
21. Fourie CM, Van Rooyen JM, Kruger A, Schutte AE. Lipid abnormalities in a never-treated HIV-1 subtype C-infected African population. Lipids. 2010;45: 73-80.
22. Freitas P, Carvalho D, Souto S, Santos AC, Xerinda S, Marques R, et al. Impact of Lipodystrophy on the prevalence and components of metabolic syndrome in HIV-infected patients. BMC infectious diseases. 2011;11: 246-2334-2311-2246.
23. Gasparotto AS, Sprinz E, Lazzaretti RK, Kuhmmer R, Silveira JM, Basso RP, et al. Genetic polymorphisms in estrogen receptors and sexual dimorphism in fat redistribution in HIV-infected patients on HAART. AIDS (London, England). 2012;26: 19-26.
24. Gazzaruso C, Bruno R, Garzaniti A, Giordanetti S, Fratino P, Sacchi P, et al. Hypertension among HIV patients: prevalence and relationships to insulin resistance and metabolic syndrome. Journal of hypertension. 2003;21: 1377-1382.
25. Guaraldi G, Beggi M, Zona S, Luzi K, Orlando G, Carli F, et al. Erectile Dysfunction Is Not a Mirror of Endothelial Dysfunction in HIV-Infected Patients. Journal of Sexual Medicine. 2012;9: 1114-1121.
26. Guaraldi G, Lonardo A, Ballestri S, Zona S, Stentarelli C, Orlando G, et al. Human immunodeficiency virus is the major determinant of steatosis and hepatitis C virus of insulin resistance in virus-associated fatty liver disease. Archives of Medical Research. 2011;42: 690-697.
27. Gupta V, Biswas A, Sharma SK. Metabolic and body composition changes after six months of highly active antiretroviral therapy in northern Indian patients. International Journal of STD & AIDS. 2011;22: 46-49.
28. Hadigan C, Edwards E, Rosenberg A, Purdy JB, Fleischman E, Howard L, et al. Microalbuminuria in HIV disease. American Journal of Nephrology. 2013;37: 443-451.
29. Hansen BR, Petersen J, Haugaard SB, Madsbad S, Obel N, Suzuki Y, et al. The prevalence of metabolic syndrome in Danish patients with HIV infection: The effect of antiretroviral therapy. HIV Medicine. 2009;10: 378-387.
30. Jacobson DL, Tang AM, Spiegelman D, Thomas AM, Skinner S, Gorbach SL, et al. Incidence of metabolic syndrome in a cohort of HIV-infected adults and prevalence relative to the US population (National Health and Nutrition Examination Survey). Journal of acquired immune deficiency syndromes (1999). 2006;43: 458-466.

31. Janiszewski PM, Ross R, Despres JP, Lemieux I, Orlando G, Carli F, et al. Hypertriglyceridemia and waist circumference predict cardiovascular risk among HIV patients: a cross-sectional study. PLoS One. 2011;6: e25032.
32. Jantarapakde J, Phanuphak N, Chaturawit C, Pengnonyang S, Mathajittiphan P, Takamtha P, et al. Prevalence of metabolic syndrome among antiretroviral-naive and antiretroviral-experienced HIV-1 infected thai adults. AIDS Patient Care and STDs. 2014;28: 331-340.
33. Jerico C, Knobel H, Montero M, Ordonez-Llanos J, Guelar A, Gimeno JL, et al. Metabolic syndrome among HIV-infected patients: prevalence, characteristics, and related factors. Diabetes Care. 2005;28: 132-137.
34. Johnsen S, Dolan SE, Fitch KV, Kanter JR, Hemphill LC, Connelly JM, et al. Carotid intimal medial thickness in human immunodeficiency virus-infected women: effects of protease inhibitor use, cardiac risk factors, and the metabolic syndrome. The Journal of clinical endocrinology and metabolism. 2006;91: 4916-4924.
35. Krishnan S, Schouten JT, Atkinson B, Brown T, Wohl D, McComsey GA, et al. Metabolic syndrome before and after initiation of antiretroviral therapy in treatment-naive HIV-infected individuals. Journal of acquired immune deficiency syndromes (1999). 2012;61: 381-389.
36. Lauda LG, Mariath AB, Grillo LP. Metabolic syndrome and its components in HIV-infected individuals. Revista da Associacao Medica Brasileira (1992). 2011;57: 182-186.
37. Leite LHM, Sampaio ABMM. Dietary calcium, dairy food intake and metabolic abnormalities in HIV-infected individuals. Journal of Human Nutrition & Dietetics. 2010;23: 535-543.
38. Maloberti A, Giannattasio C, Dozio D, Betelli M, Villa P, Nava S, et al. Metabolic syndrome in human immunodeficiency virus-positive subjects: prevalence, phenotype, and related alterations in arterial structure and function. Metabolic syndrome and related disorders. 2013;11: 403-411.
39. Martin Lde S, Pasquier E, Roudaut N, Vandhuick O, Vallet S, Bellein V, et al. Metabolic syndrome: a major risk factor for atherosclerosis in HIV-infected patients (SHIVA study). Presse medicale (Paris, France : 1983). 2008;37: 579-584.
40. Mbunkah HA, Meriki HD, Kukwah AT, Nfor O, Nkuo-Akenji T. Prevalence of metabolic syndrome in human immunodeficiency virus - infected patients from the South-West region of Cameroon, using the adult treatment panel III criteria. Diabetology & metabolic syndrome. 2014;6: 92-5996-5996-5992. eCollection 2014.

41. Mondy K, Overton ET, Grubb J, Tong S, Seyfried W, Powderly W, et al. Metabolic syndrome in HIV-infected patients from an urban, midwestern US outpatient population. Clinical infectious diseases : an official publication of the Infectious Diseases Society of America. 2007;44: 726-734.
42. Muhammad S, Sani MU, Okeahialam BN. Cardiovascular disease risk factors among HIV-infected Nigerians receiving highly active antiretroviral therapy. Nigerian medical journal : journal of the Nigeria Medical Association. 2013;54: 185-190.
43. Palacios R, Santos J, Gonzalez M, Ruiz J, Marquez M. Incidence and prevalence of the metabolic syndrome in a cohort of naive HIV-infected patients: prospective analysis at 48 weeks of highly active antiretroviral therapy. International Journal of STD & AIDS. 2007;18: 184-187.
44. Ngatchou W, Lemogoum D, Ndobo P, Yagnigni E, Tiogou E, Nga E, et al. Increased burden and severity of metabolic syndrome and arterial stiffness in treatment-naive HIV+ patients from Cameroon. Vascular health and risk management. 2013;9: 509-516.
45. Pullinger CR, Aouizerat BE, Gay C, Coggins T, Movsesyan I, Davis H, et al. Metabolic abnormalities and coronary heart disease risk in human immunodeficiency virus-infected adults. Metabolic syndrome and related disorders. 2010;8: 279-286.
46. Ramírez-Marrero FA, De Jesuś E, Santana-Bagur J, Hunter R, Frontera W, Joyner MJ. Prevalence of cardiometabolic risk factors in hispanics living with HIV. Ethnicity and Disease. 2010;20: 423-428.
47. Samaras K, Wand H, Matthew L, Emery S, Cooper D, Carr A. Prevalence of metabolic syndrome in HIV-infected patients receiving highly active antiretroviral therapy using International Diabetes Foundation and Adult Treatment Panel III criteria: Associations with insulin resistance, disturbed body fat compartmentalization, elevated C-reactive protein, and hypoadiponectinemia. Diabetes care. 2007;30: 113-119.
48. Sawadogo A, Sanou S, Hema A, Kamboule BE, Kabore NF, Sore I, et al. Metabolic syndrome and cardiovascular risk patients under antiretrovirals in a hospital day at Bobo-Dioulasso (Burkina Faso). Bulletin de la Societe de Pathologie Exotique. 2014;107: 151-158.
49. Schillaci G, De Socio GV, Pucci G, Mannarino MR, Helou J, Pirro M, et al. Aortic stiffness in untreated adult patients with human immunodeficiency virus infection. Hypertension. 2008;52: 308-313.
50. Signorini DJHP, Monteiro MCM, de Andrade MFC, Signorini DH, Eyer-Silva WA. What should we know about metabolic syndrome and lipodystrophy in AIDS? Revista da Associacao Medica Brasileira. 2012;58: 70-75.
51. Sobieszczyk ME, Hoover DR, Anastos K, Mulligan K, Tan T, Shi Q, et al. Prevalence and predictors of metabolic syndrome among HIV-infected and HIV-uninfected women in the women's interagency HIV Study. Journal of acquired immune deficiency syndromes. 2008;48: 272-280.

52. Sterling RK, Contos MJ, Smith PG, Stravitz RT, Luketic VA, Fuchs M, et al. Steatohepatitis: Risk factors and impact on disease severity in human immunodeficiency virus/hepatitis C virus coinfection. Hepatology. 2008;47: 1118-1127.

53. Tesfaye DY, Kinde S, Medhin G, Megerssa YC, Tadewos A, Tadesse E, et al. Burden of metabolic syndrome among HIV-infected patients in Southern Ethiopia. Diabetes & metabolic syndrome. 2014;8: 102-107.

54. Tiozzo E, Konefal J, Adwan S, Martinez LA, Villabona J, Lopez J, et al. A cross-sectional assessment of metabolic syndrome in HIV-infected people of low socio-economic status receiving antiretroviral therapy

Changes in metabolic syndrome status after initiation of antiretroviral therapy. Diabetol Metab Syndr. 2015;7: 15.

55. Wand H, Calmy A, Carey DL, Samaras K, Carr A, Law MG, et al. Metabolic syndrome, cardiovascular disease and type 2 diabetes mellitus after initiation of antiretroviral therapy in HIV infection. AIDS (London, England). 2007;21: 2445-2453.

56. Worm SW, Friis-Moller N, Bruyand M, D'Arminio Monforte A, Rickenbach M, Reiss P, et al. High prevalence of the metabolic syndrome in HIV-infected patients: impact of different definitions of the metabolic syndrome. AIDS (London, England). 2010;24: 427-435.

57. Wu PY, Hung CC, Liu WC, Hsieh CY, Sun HY, Lu CL, et al. Metabolic syndrome among HIV-infected Taiwanese patients in the era of highly active antiretroviral therapy: Prevalence and associated factors. Journal of Antimicrobial Chemotherapy. 2012;67: 1001-1009.

58. Young J, Glass TR, Bernasconi E, Rickenbach M, Furrer H, Hirschel B, et al. Hierarchical modeling gave plausible estimates of associations between metabolic syndrome and components of antiretroviral therapy. Journal of clinical epidemiology. 2009;62: 632-641.

59. Zannou DM, Denoeud L, Lacombe K, Amoussou-Guenou D, Bashi J, Akakpo J, et al. Incidence of lipodystrophy and metabolic disorders in patients starting non-nucleoside reverse transcriptase inhibitors in Benin. Antiviral therapy. 2009;14: 371-380.
