# Supplementary material for: A Meta-Analysis of the Metabolic Syndrome Prevalence in the Global HIV-Infected Population
Source: PLoS One. 2016 Mar 23;11(3):e0150970. doi: 10.1371/journal.pone.0150970 (PMC4805252; doi:10.1371/journal.pone.0150970)
Supplement: S5 Table — (PDF) [file pone.0150970.s008.pdf]

S5 Table. The prevalence of metabolic syndrome estimated by the included studies according to criteria

**Reference**	**Country**	**Area**	**Study site**	**Study type**	**Study period**	**N**	**Overall prevalence of metabolic syndrome**						
							EGIR 2003	ATPIII 2001	ATPIII 2004-2005	IDF 2005	JIS 2009	Modified ATPIII 2005	* Modifications noted
	**Intercontinental**												
Samaras, et al, 2007 [1]	USA, Europe, Australia, Asia, South America	National	Hospital + community based	Case-control	NR	788		18		14			
Wand, et al, 2007 [2]	Australia, Brazil, Canada, New Zealand, 17 European countries	Urban	Hospital	Cross-sectional	1999-2002	881		8.5		7.8			
Worm, et al, 2010 [3]	USA, Australia, 21 European countries	National	Hospital (212 clinics)	Cross-sectional	2006-2007	23853						41.6*	Adapted ATPIII 2005, using BMI ≥30 for central obesity component
	**America**												
Adeyemi, et al, 2008 [4]	USA	Urban	Hospital	Cross-sectional	2005-2006	121			34				
Ances, et al, 2009 [5]	USA	National	Hospital	Case-control	NR	66						15.2*	Adapted ATPIII 2005, using BMI for central obesity
Baum, et al, 2006 [6]	USA	National	Community	Cross-sectional	2002-2003	118		15.1					
Hadigan, et al, 2013 [7]	USA	Urban	Hospital (2 clinics)	Cross-sectional	2007-2011	182				13.7			
Jacobson, et al, 2006 [8]	USA	Urban	Community	Cross-sectional	2000-2003	477			24				
Johnsen, et al, 2006 [9]	USA	National	Hospital	Case-control	2002-2003	97		31					
Krishnan, et al, 2012 [10]	USA	National	Hospital	Cross-sectional	2001-2007	2247			20				
Mondy, et al, 2007 [11]	USA	Urban	Hospital	Cross-sectional	2005	471			25.5				
Pullinger, et al, 2010 [12]	USA	Urban	Community	Longitudinal, cross-sectional	2005-2007	296		30					
Sobieszczyk, et al, 2008[13]	USA	Urban	Hospital + community	Prospective, cross-sectional	2000-2004	1725			33				
Sterling, et al, 2008 [14]	USA	Urban	Hospital	Cross-sectional	1998-2006	222		9					

Tiozzo, et al, 2015 [15]	USA	Urban	Hospital	Cross-sectional	2013	89	33*				Adapted ATPIII 2001, including taking treatments for hypertension or diabetes mellitus
Cahn, et al, 2010 [16]	Argentina	National	Hospital	Cross-sectional	2006-2007	1015	22.3				
	Brazil	National	Hospital	Cross-sectional	2006-2007	1001	25.4				
	Colombia	National	Hospital	Cross-sectional	2006-2007	474	8.4				
	Chile	National	Hospital	Cross-sectional	2006-2007	44	13.6				
	Ecuador	National	Hospital	Cross-sectional	2006-2007	252	19.1				
	Peru	National	Hospital	Cross-sectional	2006-2007	417	13.7				
	Venezuela	National	Hospital	Cross-sectional	2006-2007	807	22.4				
Alencastro, et al, 2012 [17]	Brazil	Urban	Hospital	Cross-sectional	NR	1240		17.2	22.1	24.7	
Da Silva, et al, 2009 [18]	Brazil	Urban	Hospital (7 centers)	Cross-sectional	2004-2006	319	12.3				
Gasparotto, et al, 2012 [19]	Brazil	National	Hospital (multi-centers)	Cross-sectional	NR	614	19.4		31.9		
Lauda, et al, 2011 [20]	Brazil	Urban	Hospital	Cross-sectional	2007-2008	249	20.9				
Leite, et al, 2010 [21]	Brazil	Urban	Hospital	Cross-sectional	2008	100		52			
Signorini, et al, 2012 [22]	Brazil	National	Hospital	Cross-sectional	2005	819	20.6				
Ramirez-Marrero, et al, 2010 [23]	Puerto Rico	Urban	Hospital community	Cross-sectional	2003-2007	897		35.4			
	**Europe**										
Hansen, et al, 2009 [24]	Denmark	National	Hospital	Cross-sectional	2004-2006	566	27				
Badiou, et al, 2008 [25]	France	National	Hospital	Cross-sectional	1999	232	7.3		11.2		
Biron, et al, 2012 [26]	France	National	Hospital (5 centers)	Cross-sectional	2000-2007	269		18.2			
Martin, et al, 2008 (SHIVA study) [27]	France	Urban	Hospital	Cross-sectional	2003	140	7.1				
Sawadogo, et al, 2014 [28]	Burkina Faso	Urban	Hospital	Cross-sectional	2011	400		12.3	10		

Bonfanti, et al, 2010 [29]	Italy	Urban	Hospital (14 centers)	Cross-sectional	2007	292		12.3*				Adapted ATPIII 2001, including taking medication for HTN or DM
Bonfanti, et al, 2007[30]	Italy	Urban	Hospital (18 centers)	Cross-sectional	2005	1243		20.8*				Adapted ATPIII 2001, including taking medication for HTN or DM
Calza, et al, 2011 [31]	Italy	Urban	Hospital	Cross-sectional	2009	755		9.1				
De Socio, et al, 2014 (HIV-Hy study) [32]	Italy	National	Hospital	Cross-sectional	2010-2011	765					33.4	
Gazzaruso, et al, 2003 [33]	Italy	National	Hospital	Cross-sectional	NR	287	33.1					
Guaraldi, et al, 2012 [34]	Italy	National	Hospital (2 centers)	Cross-sectional	2009-2010	133			24.8			
Guaraldi, et al, 2011 [35]	Italy	National	Hospital (2 centers)	Cross-sectional	2007-2008	143		14.7				
Janiszewsk,i et al, 2011 [36]	Italy	National	Hospital	Cross-sectional	2005-2009	2322		25.6				
Maloberti, et al, 2013[37]	Italy	National	Hospital	Cross-sectional	NR	108		17.6*				Adapted ATPIII 2001, using BMI levels corresponding with WC in Italian adults
Schillaci, et al, 2008 [38]	Italy	Urban	Hospital	Case-control	NR	39		18				
Bergersen,et al, 2006 [39]	Norway	Urban	Hospital	Cross-sectional	2000-2001	263		13.3*				Adapted ATPIII 2001, including taking medications for HTN or DM
Freitas, et al, 2011 [40]	Portugal	National	Hospital	Cross-sectional	NR	345			52.2	43.2		
Cubero, et al, 2011 [41]	Spain	National	Hospital	Cross-sectional	NR	159	28.3	10.1		15.1		
Estrada, et al, 2006 [42]	Spain	National	Hospital	Cross-sectional	NR	146			15.8			
Jerico, et al, 2005 [43]	Spain	Urban	Hospital	Cross-sectional	2003	710		17*				Adapted ATPIII 2001, including taking medications for HTN or DM
Palacios, et al, 2007 [44]	Spain	National	Hospital	Cross-sectional	2002-2003	60		16.6*				Adapted ATPIII 2001, using BMI≥26.7 (validated) for central obesity

Young, et al, 2009 [45]	Switzerland	National	Hospital	Cross-sectional	2000-2006	1644			15*		Adapted IDF 2005, using random glucose level for raised glucoism
Elgalib, et al, 2011 [46]	UK	Urban; Peri-urban	Hospital (2 centers)	Cross-sectional	2005-2006	678		14	10		
	**Africa**										
Zannou, et al, 2009 [47]	Benin	Urban	Hospital	Cross-sectional	2004-2005	79			13		
Mbunkah, et al, 2014 [48]	Cameroon	National	Hospital	Cross-sectional	2010-2011	173	15.6				
Ngatchou, et al, 2013 [49]	Cameroon	Urban	Hospital	Cross-sectional	2009-2010	108				47	
Berhane, et al, 2012 [50]	Ethiopia	Urban	Hospital	Cross-sectional	2010	313		21.1			
Tesfaye, et al, 2014 [51]	Ethiopia	Urban	Hospital	Cross-sectional	2013	374	16.8		23.8		
Ayodele, et al, 2012 [52]	Nigeria	Urban	Hospital	Cross-sectional	NR	291	12.7		17.2	21	
Muhammad, et al, 2013 [53]	Nigeria	Urban	Hospital	Cross-sectional	2009	200	15				
Awotedu, et al, 2010 [54]	South Africa	Urban	Hospital	Cross-sectional	2009-2010	196	20.4		22.9		
Fourie, et al, 2010 [55]	South Africa	Urban; Rural	Community	Cross-sectional	2005	300	15.2		21.1		
	**Asia**										
Gupta, et al, 2011 [56]	India	Urban	Hospital	Cross-sectional	2007-2009	68		25	19.1		
Bajaj, et al, 2013 [57]	India	Urban	Hospital	Cross-sectional	2010-2011	70		20			
Wu, et al, 2012 [58]	Taiwan	Nationwide	Hospital	Cross-sectional	2008-2009	803		26.2			
Jantarapak,de et al, 2014 [59]	Thailand	National	Hospital (6 centers)	Cross-sectional	2009-2011	580		22.2			

ATPIII, Adults Treatment Panel III; BMI, body mass index; DM, diabetes mellitus; EGIR, European Group for the Study of Insulin Resistance; HTN, hypertension; IDF, International Diabetes Federation; JIS, Joint Interim Statement; NR, not reported; WC, waist circumference

References

1. Samaras K, Wand H, Matthew L, Emery S, Cooper D, Carr A. Prevalence of metabolic syndrome in HIV-infected patients receiving highly active antiretroviral therapy using International Diabetes Foundation and Adult Treatment Panel III criteria: Associations with insulin resistance, disturbed body fat compartmentalization, elevated C-reactive protein, and hypoadiponectinemia. Diabetes care. 2007;30: 113-119.
2. Wand H, Calmy A, Carey DL, Samaras K, Carr A, Law MG, et al. Metabolic syndrome, cardiovascular disease and type 2 diabetes mellitus after initiation of antiretroviral therapy in HIV infection. AIDS (London, England). 2007;21: 2445-2453.
3. Worm SW, Friis-Moller N, Bruyand M, D'Arminio Monforte A, Rickenbach M, Reiss P, et al. High prevalence of the metabolic syndrome in HIV-infected patients: impact of different definitions of the metabolic syndrome. AIDS (London, England). 2010;24: 427-435.
4. Adeyemi O, Rezai K, Bahk M, Badri S, Thomas-Gossain N. Metabolic syndrome in older HIV-infected patients: data from the CORE50 cohort. AIDS Patient Care and STDs. 2008;22: 941-945.
5. Ances BM, Bhatt A, Vaida F, Rosario D, Alexander T, Marquie-Beck J, et al. Role of metabolic syndrome components in human immunodeficiency virus-associated stroke. Journal of neurovirology. 2009;15: 249-256.
6. Baum MK, Rafie C, Lai S, Xue L, Sales S, Page JB, et al. Coronary Heart Disease (CHD) Risk Factors and Metabolic Syndrome in HIV-Positive Drug Users in Miami. American journal of infectious diseases. 2006;2: 173-179.
7. Hadigan C, Edwards E, Rosenberg A, Purdy JB, Fleischman E, Howard L, et al. Microalbuminuria in HIV disease. American Journal of Nephrology. 2013;37: 443-451.
8. Jacobson DL, Tang AM, Spiegelman D, Thomas AM, Skinner S, Gorbach SL, et al. Incidence of metabolic syndrome in a cohort of HIV-infected adults and prevalence relative to the US population (National Health and Nutrition Examination Survey). Journal of acquired immune deficiency syndromes (1999). 2006;43: 458-466.
9. Johnsen S, Dolan SE, Fitch KV, Kanter JR, Hemphill LC, Connelly JM, et al. Carotid intimal medial thickness in human immunodeficiency virus-infected women: effects of protease inhibitor use, cardiac risk factors, and the metabolic syndrome. The Journal of clinical endocrinology and metabolism. 2006;91: 4916-4924.

10. Krishnan S, Schouten JT, Atkinson B, Brown T, Wohl D, McComsey GA, et al. Metabolic syndrome before and after initiation of antiretroviral therapy in treatment-naive HIV-infected individuals. Journal of acquired immune deficiency syndromes (1999). 2012;61: 381-389.

11. Mondy K, Overton ET, Grubb J, Tong S, Seyfried W, Powderly W, et al. Metabolic syndrome in HIV-infected patients from an urban, midwestern US outpatient population. Clinical infectious diseases : an official publication of the Infectious Diseases Society of America. 2007;44: 726-734.

12. Pullinger CR, Aouizerat BE, Gay C, Coggins T, Movsesyan I, Davis H, et al. Metabolic abnormalities and coronary heart disease risk in human immunodeficiency virus-infected adults. Metabolic syndrome and related disorders. 2010;8: 279-286.

13. Sobieszczyk ME, Hoover DR, Anastos K, Mulligan K, Tan T, Shi Q, et al. Prevalence and predictors of metabolic syndrome among HIV-infected and HIV-uninfected women in the women's interagency HIV Study. Journal of acquired immune deficiency syndromes. 2008;48: 272-280.

14. Sterling RK, Contos MJ, Smith PG, Stravitz RT, Luketic VA, Fuchs M, et al. Steatohepatitis: Risk factors and impact on disease severity in human immunodeficiency virus/hepatitis C virus coinfection. Hepatology. 2008;47: 1118-1127.

15. Tiozzo E, Konefal J, Adwan S, Martinez LA, Villabona J, Lopez J, et al. A cross-sectional assessment of metabolic syndrome in HIV-infected people of low socio-economic status receiving antiretroviral therapy

Changes in metabolic syndrome status after initiation of antiretroviral therapy. Diabetol Metab Syndr. 2015;7: 15.

16. Cahn P, Leite O, Rosales A, Cabello R, Alvarez CA, Seas C, et al. Metabolic profile and cardiovascular risk factors among Latin American HIV-infected patients receiving HAART. The Brazilian journal of infectious diseases : an official publication of the Brazilian Society of Infectious Diseases. 2010;14: 158-166.

17. Alencastro PR, Wolff FH, Oliveira RR, Ikeda ML, Barcellos NT, Brandao AB, et al. Metabolic syndrome and population attributable risk among HIV/AIDS patients: comparison between NCEP-ATPIII, IDF and AHA/NHLBI definitions. AIDS research and therapy. 2012;9: 29-6405-6409-6429.

18. Da Silva ÉFR, Bassichetto KC, Lewi DS. Lipid profile, cardiovascular risk factors and metabolic syndrome in a group of AIDS patients. Arquivos Brasileiros de Cardiologia. 2009;93: 113-118.

19. Gasparotto AS, Sprinz E, Lazzaretti RK, Kuhmmer R, Silveira JM, Basso RP, et al. Genetic polymorphisms in estrogen receptors and sexual dimorphism in fat redistribution in HIV-infected patients on HAART. AIDS (London, England). 2012;26: 19-26.

20. Lauda LG, Mariath AB, Grillo LP. Metabolic syndrome and its components in HIV-infected individuals. Revista da Associacao Medica Brasileira (1992). 2011;57: 182-186.

21. Leite LHM, Sampaio ABMM. Dietary calcium, dairy food intake and metabolic abnormalities in HIV-infected individuals. Journal of Human Nutrition & Dietetics. 2010;23: 535-543.

22. Signorini DJHP, Monteiro MCM, de Andrade MFC, Signorini DH, Eyer-Silva WA. What should we know about metabolic syndrome and lipodystrophy in AIDS? Revista da Associacao Medica Brasileira. 2012;58: 70-75.

23. Ramírez-Marrero FA, De Jesuś E, Santana-Bagur J, Hunter R, Frontera W, Joyner MJ. Prevalence of cardiometabolic risk factors in hispanics living with HIV. Ethnicity and Disease. 2010;20: 423-428.

24. Hansen BR, Petersen J, Haugaard SB, Madsbad S, Obel N, Suzuki Y, et al. The prevalence of metabolic syndrome in Danish patients with HIV infection: The effect of antiretroviral therapy. HIV Medicine. 2009;10: 378-387.

25. Badiou S, Thiebaut R, Aurillac-Lavignolle V, Dabis F, Laporte F, Cristol JP, et al. Association of non-HDL cholesterol with subclinical atherosclerosis in HIV-positive patients. The Journal of infection. 2008;57: 47-54.

26. Biron A, Bobin-Dubigeon C, Volteau C, Piroth L, Perre P, Leport C, et al. Metabolic syndrome in French HIV-infected patients: prevalence and predictive factors after 3 years of antiretroviral therapy. AIDS Research and Human Retroviruses. 2012;28: 1672-1678.

27. Martin Lde S, Pasquier E, Roudaut N, Vandhuick O, Vallet S, Bellein V, et al. Metabolic syndrome: a major risk factor for atherosclerosis in HIV-infected patients (SHIVA study). Presse medicale (Paris, France : 1983). 2008;37: 579-584.

28. Sawadogo A, Sanou S, Hema A, Kamboule BE, Kabore NF, Sore I, et al. Metabolic syndrome and cardiovascular risk patients under antiretrovirals in a hospital day at Bobo-Dioulasso (Burkina Faso). Bulletin de la Societe de Pathologie Exotique. 2014;107: 151-158.

29. Bonfanti P, De Socio GL, Marconi P, Franzetti M, Martinelli C, Vichi F, et al. Is metabolic syndrome associated to HIV infection per se? Results from the HERMES study. Current HIV research. 2010;8: 165-171.

30. Bonfanti P, Giannattasio C, Ricci E, Facchetti R, Rosella E, Franzetti M, et al. HIV and metabolic syndrome: a comparison with the general population. Journal of acquired immune deficiency syndromes (1999). 2007;45: 426-431.

31. Calza L, Masetti G, Piergentili B, Trapani F, Cascavilla A, Manfredi R, et al. Prevalence of diabetes mellitus, hyperinsulinaemia and metabolic syndrome among 755 adult patients with HIV-1 infection. International Journal of STD and AIDS. 2011;22: 43-45.
32. De Socio GV, Ricci E, Maggi P, Parruti G, Pucci G, Di Biagio A, et al. Prevalence, awareness, treatment, and control rate of hypertension in HIV-infected patients: The HIV-HY study. American Journal of Hypertension. 2014;27: 222-228.
33. Gazzaruso C, Bruno R, Garzaniti A, Giordanetti S, Fratino P, Sacchi P, et al. Hypertension among HIV patients: prevalence and relationships to insulin resistance and metabolic syndrome. Journal of hypertension. 2003;21: 1377-1382.
34. Guaraldi G, Beggi M, Zona S, Luzi K, Orlando G, Carli F, et al. Erectile Dysfunction Is Not a Mirror of Endothelial Dysfunction in HIV-Infected Patients. Journal of Sexual Medicine. 2012;9: 1114-1121.
35. Guaraldi G, Lonardo A, Ballestri S, Zona S, Stentarelli C, Orlando G, et al. Human immunodeficiency virus is the major determinant of steatosis and hepatitis C virus of insulin resistance in virus-associated fatty liver disease. Archives of Medical Research. 2011;42: 690-697.
36. Janiszewski PM, Ross R, Despres JP, Lemieux I, Orlando G, Carli F, et al. Hypertriglyceridemia and waist circumference predict cardiovascular risk among HIV patients: a cross-sectional study. PLoS One. 2011;6: e25032.
37. Maloberti A, Giannattasio C, Dozio D, Betelli M, Villa P, Nava S, et al. Metabolic syndrome in human immunodeficiency virus-positive subjects: prevalence, phenotype, and related alterations in arterial structure and function. Metabolic syndrome and related disorders. 2013;11: 403-411.
38. Schillaci G, De Socio GV, Pucci G, Mannarino MR, Helou J, Pirro M, et al. Aortic stiffness in untreated adult patients with human immunodeficiency virus infection. Hypertension. 2008;52: 308-313.
39. Bergersen BM, Schumacher A, Sandvik L, Bruun JN, Birkeland K. Important differences in components of the metabolic syndrome between HIV-patients with and without highly active antiretroviral therapy and healthy controls. Scandinavian Journal of Infectious Diseases. 2006;38: 682-689.
40. Freitas P, Carvalho D, Souto S, Santos AC, Xerinda S, Marques R, et al. Impact of Lipodystrophy on the prevalence and components of metabolic syndrome in HIV-infected patients. BMC infectious diseases. 2011;11: 246-2334-2311-2246.
41. Cubero JM, Domingo P, Sambeat M, Ordoñez-Llanos J, Rodriguez-Espinosa J, Sánchez-Quesada JL, et al. Prevalence of metabolic syndrome among human immunodeficiency virus-infected subjects is widely influenced by the diagnostic criteria. Metabolic Syndrome and Related Disorders. 2011;9: 345-351.

42. Estrada V, Martinez-Larrad MT, Gonzalez-Sanchez JL, de Villar NG, Zabena C, Fernandez C, et al. Lipodystrophy and metabolic syndrome in HIV-infected patients treated with antiretroviral therapy. Metabolism: clinical and experimental. 2006;55: 940-945.
43. Jerico C, Knobel H, Montero M, Ordonez-Llanos J, Guelar A, Gimeno JL, et al. Metabolic syndrome among HIV-infected patients: prevalence, characteristics, and related factors. Diabetes Care. 2005;28: 132-137.
44. Palacios R, Santos J, Gonzalez M, Ruiz J, Marquez M. Incidence and prevalence of the metabolic syndrome in a cohort of naive HIV-infected patients: prospective analysis at 48 weeks of highly active antiretroviral therapy. International Journal of STD & AIDS. 2007;18: 184-187.
45. Young J, Glass TR, Bernasconi E, Rickenbach M, Furrer H, Hirschel B, et al. Hierarchical modeling gave plausible estimates of associations between metabolic syndrome and components of antiretroviral therapy. Journal of clinical epidemiology. 2009;62: 632-641.
46. Elgalib A, Aboud M, Kulasegaram R, Dimian C, Duncan A, Wierzbicki AS, et al. The assessment of metabolic syndrome in UK patients with HIV using two different definitions: CREATE 2 study. Current medical research and opinion. 2011;27: 63-69.
47. Zannou DM, Denoeud L, Lacombe K, Amoussou-Guenou D, Bashi J, Akakpo J, et al. Incidence of lipodystrophy and metabolic disorders in patients starting non-nucleoside reverse transcriptase inhibitors in Benin. Antiviral therapy. 2009;14: 371-380.
48. Mbunkah HA, Meriki HD, Kukwah AT, Nfor O, Nkuo-Akenji T. Prevalence of metabolic syndrome in human immunodeficiency virus - infected patients from the South-West region of Cameroon, using the adult treatment panel III criteria. Diabetology & metabolic syndrome. 2014;6: 92-5996-5996-5992. eCollection 2014.
49. Ngatchou W, Lemogoum D, Ndobo P, Yagnigni E, Tiogou E, Nga E, et al. Increased burden and severity of metabolic syndrome and arterial stiffness in treatment-naive HIV+ patients from Cameroon. Vascular health and risk management. 2013;9: 509-516.
50. Berhane T, Yami A, Alemseged F, Yemane T, Hamza L, Kassim M, et al. Prevalence of lipodystrophy and metabolic syndrome among HIV positive individuals on highly active anti-retroviral treatment in Jimma, south west Ethiopia. Pan African Medical Journal. 2012;13.
51. Tesfaye DY, Kinde S, Medhin G, Megerssa YC, Tadewos A, Tadesse E, et al. Burden of metabolic syndrome among HIV-infected patients in Southern Ethiopia. Diabetes & metabolic syndrome. 2014;8: 102-107.

52. Ayodele OE, Akinboro AO, Akinyemi SO, Adepeju AA, Akinremi OA, Alao CA, et al. Prevalence and clinical correlates of metabolic syndrome in Nigerians living with human immunodeficiency virus/acquired immunodeficiency syndrome. Metabolic syndrome and related disorders. 2012;10: 373-379.
53. Muhammad S, Sani MU, Okeahialam BN. Cardiovascular disease risk factors among HIV-infected Nigerians receiving highly active antiretroviral therapy. Nigerian medical journal : journal of the Nigeria Medical Association. 2013;54: 185-190.
54. Awotedu K, Ekpebegh C, Longo-Mbenza B, Iputo J. Prevalence of metabolic syndrome assessed by IDF and NCEP ATP 111 criteria and determinants of insulin resistance among HIV patients in the Eastern Cape Province of South Africa. Diabetes and Metabolic Syndrome: Clinical Research and Reviews. 2010;4: 210-214.
55. Fourie CM, Van Rooyen JM, Kruger A, Schutte AE. Lipid abnormalities in a never-treated HIV-1 subtype C-infected African population. Lipids. 2010;45: 73-80.
56. Gupta V, Biswas A, Sharma SK. Metabolic and body composition changes after six months of highly active antiretroviral therapy in northern Indian patients. International Journal of STD & AIDS. 2011;22: 46-49.
57. Bajaj S, Tyagi SK, Bhargava A. Metabolic syndrome in human immunodeficiency virus positive patients. Indian journal of endocrinology and metabolism. 2013;17: 117-120.
58. Wu PY, Hung CC, Liu WC, Hsieh CY, Sun HY, Lu CL, et al. Metabolic syndrome among HIV-infected Taiwanese patients in the era of highly active antiretroviral therapy: Prevalence and associated factors. Journal of Antimicrobial Chemotherapy. 2012;67: 1001-1009.
59. Jantarapakde J, Phanuphak N, Chaturawit C, Pengnonyang S, Mathajittiphan P, Takamtha P, et al. Prevalence of metabolic syndrome among antiretroviral-naive and antiretroviral-experienced HIV-1 infected thai adults. AIDS Patient Care and STDs. 2014;28: 331-340.
