# Supplementary material for: A Meta-Analysis of the Metabolic Syndrome Prevalence in the Global HIV-Infected Population
Source: PLoS One. 2016 Mar 23;11(3):e0150970. doi: 10.1371/journal.pone.0150970 (PMC4805252; doi:10.1371/journal.pone.0150970)
Supplement: S6 Table — (PDF) [file pone.0150970.s009.pdf]

S6 Table. Summary statistics from meta-analyses of prevalence studies on metabolic syndrome in people with HIV using random effects model and arcsine transformations

Group	Subgroup	Criteria	N studies	N participants	N Cases	Prevalence (95% CI)	H (95% CI)	I^2 (95% CI)	p-heterogeneity	P-dif criteria	p-diff sub-groups	p-Egger test
Overall										0.001		
		ATPIII2001	38	16984	3179	16.7 [14.6-18.8]	3.56 [3.18-3.99]	92.1 [90.1-93.7]	<0.001			0.040
		ATPIII2004-2005	20	11255	2768	24.6 [20.6-28.8]	4.90 [4.30-5.60]	95.8 [94.6-96.8]	<0.001			0.870
		IDF2005	16	8250	1471	18.0 [14.0-22.4]	4.90 [4.20-5.60]	95.8 [94.4-96.9]	<0.001			0.610
		JIS2009	4	2404	654	29.6 [22.9-36.8]	3.40 [2.30-5.00]	91.0 [80.6-96.0]	<0.001			0.490
		Modified ATPIII2005	2	23919	9923	27.9 [06.7-56.5]	4.90 [-]	95.8 [-]	<0.001			-
		EGIR2003	2	446	140	31.3 [26.8-36.0]	1.10 [-]	09.8 [-]	0.300			-
Gender	Overall									0.001		
		ATPIII2001	33	12240	2299	16.2 [13.8-18.4]	3.26 [2.87-3.71]	90.6 [87.9-92.7]	<0.001		0.250	0.140
		ATPIII2004-2005	26	10212	2523	25.1 [21.3-29.1]	4.36 [3.86-4.92]	94.7 [93.3-95.9]	<0.001		0.450	0.710
		IDF2005	22	5849	1088	17.9 [13.6-22.7]	4.50 [3.96-5.12]	95.1 [93.6-96.2]	<0.001		0.030	0.710
	Men									0.003		
		ATPIII2001	16	8269	1530	14.6 [11.5-18.1]	3.82 [3.22-4.52]	93.1 [90.4-95.1]	<0.001			0.060
		ATPIII2004-2005	13	5742	1262	23.7 [19.0-28.7]	4.15 [3.47-4.96]	94.2 [91.7-95.9]	<0.001			0.290
		IDF2005	11	3556	553	13.4 [08.7-18.9]	4.30 [3.55-5.22]	94.6 [92.1-96.3]	<0.001			0.750
	Women									0.118		
		ATPIII2001	17	3971	769	17.5 [14.0-21.2]	2.87 [2.31-3.42]	86.7 [82.6-90.5]	<0.001			0.780
		ATPIII2004-2005	13	4470	1261	26.7 [20.8-33.0]	4.26 [3.57-5.09]	94.5 [92.2-96.1]	<0.001			0.560
		IDF2005	11	2293	535	23.2 [15.9-31.4]	4.32 [3.57-5.23]	94.6 [92.1-96.3]	<0.001			0.810
Continent	Overall									0.002		
		ATPIII2001	38	16984	3179	16.7 [14.6-18.8]	3.56 [3.18-3.99]	92.1 [90.1-93.7]	<0.001		0.216	0.040
		ATPIII2004-2005	20	11255	2768	24.6 [20.6-28.8]	4.90 [4.30-5.60]	95.8 [94.6-96.8]	<0.001		0.284	0.870
		IDF2005	16	8250	1471	18.0 [14.0-22.4]	4.90 [4.20-5.60]	95.8 [94.4-96.9]	<0.001		0.100	0.610

	Intercontinental									0.748		
		ATPIII2001	2	1669	214	12.7 [05.2-22.9]	5.60 [-]	96.8 [-]	<0.001			-
		ATPIII2004-2005	-									
		IDF2005	2	1669	183	10.9 [05.3-18.2]	4.35 [-]	94.7 [-]	<0.001			-
	Americas									0.021		
		ATPIII2001	16	6798	1364	20.0 [16.0-22.2]	3.15 [2.60-3.80]	89.9 [85.3-93.1]	<0.001			0.598
		ATPIII2004-2005	9	7450	1902	27.3 [21.7-33.4]	5.39 [4.47-6.49]	96.6 [95.0-97.6]	<0.001			0.950
		IDF2005	2	1854	470	26.8 [17.8-36.9]	4.50 [-]	95.1 [-]	<0.001			-
	Europe									0.423		
		ATPIII2001	14	7032	1368	15.1 [11.5-19.1]	4.14 [3.49-4.92]	94.2 [91.8-95.9]	<0.001			0.040
		ATPIII2004-2005	5	1571	381	24.1 [11.2-40.1]	6.6 [5.24-8.32]	97.7 [96.4-98.6]	<0.001			0.770
		IDF2005	5	3058	517	18.0 [09.6-28.2]	6.15 [4.83-7.82]	97.4 [95.7-98.4]	<0.001			0.670
	Africa									0.741		
		ATPIII2001	6	1485	233	15.6 [13.8-17.5]	1.00 [1.00-1.950]	00.0 [00.0-73.6]	0.440			0.490
		ATPIII2004-2005	2	713	115	16.4 [08.7-25.9]	3.17 [-]	90.0 [-]	0.002			-
		IDF2005	6	1601	288	17.7 [12.9-23.1]	2.65 [1.86-3.78]	85.8 [71.0-93.0]	<0.001			0.910
	Asia									0.329		
		ATPIII2001	-									
		ATPIII2004-2005	4	1521	370	24.1 [21.6-26.8]	1.10 [1.00-2.80]	16.8 [00.0-87.3]	0.307			0.610
		IDF2005	1	68	13	19.1 [10.7-29.2]	-	-	-			-
Region	Overall									0.002		
		ATPIII2001	38	16984	3179	16.7 [14.6-18.8]	3.56 [3.18-3.99]	92.1 [90.1-93.7]	<0.001		0.607	0.040
		ATPIII2004-2005	20	11255	2768	24.6 [20.6-28.8]	4.90 [4.30-5.60]	95.8 [94.6-96.8]	<0.001		0.861	0.870
		IDF2005	16	8250	1471	18.0 [14.0-22.4]	4.90 [4.20-5.60]	95.8 [94.4-96.9]	<0.001		0.785	0.610
	Regional									0.024		
		ATPIII2001	16	5610	924	16.0 [13.2-19.1]	2.87 [2.35-3.50]	87.8 [81.8-91.8]	<0.001			0.870
		ATPIII2004-2005	13	6732	1694	24.3 [19.1-30.0]	4.99 [4.25-5.86]	96.0 [94.5-97.1]	<0.001			0.730
		IDF2005	9	3587	648	17.4 [13.4-21.8]	3.11 [2.40-4.03]	89.7 [82.6-93.8]	<0.001			0.802
	National									0.109		

		ATPIII2001	22	11374	2255	17.1 [14.4-20.1]	3.90 [3.39-4.49]	93.4 [91.3-95.0]	<0.001			0.060
		ATPIII2004-2005	7	4523	1074	25.1 [18.3-32.7]	5.08 [4.06-6.35]	96.1 [93.9-97.5]	<0.001			0.520
		IDF2005	7	4663	823	18.7 [11.4-27.3]	6.83 [5.67-8.22]	97.9 [96.9-98.5]	<0.001			0.503
Publication year, median 2010	Overall									0.002		
		ATPIII2001	38	16984	3179	16.7 [14.6-18.8]	3.56 [3.18-3.99]	92.1 [90.1-93.7]	<0.001		0.694	0.040
		ATPIII2004-2005	20	11255	2768	24.6 [20.6-28.8]	4.90 [4.30-5.60]	95.8 [94.6-96.8]	<0.001		0.012	0.870
		IDF2005	16	8250	1471	18.0 [14.0-22.4]	4.90 [4.20-5.60]	95.8 [94.4-96.9]	<0.001		0.101	0.607
	Above median									0.326		
		ATPIII2001	13	6296	1246	17.3 [13.7-21.2]	3.63 [2.98-4.45]	92.4 [88.8-94.8]	<0.001			0.131
		ATPIII2004-2005	13	7328	1531	21.5 [17.2-26.1]	4.40 [3.70-5.21]	94.8 [92.6-96.3]	<0.001			0.638
		IDF2005	9	4169	908	20.8 [14.4-27.9]	5.24 [4.34-6.34]	94.8 [92.6-96.3]	<0.001			0.943
	Below median									0.000		
		ATPIII2001	25	10688	1933	16.4 [13.8-19.1]	3.56 [3.10-4.09]	92.1 [89.6-94.0]	<0.001			0.214
		ATPIII2004-2005	7	3927	1237	30.6 [25.2-36.2]	3.40 [2.56-4.51]	91.3 [84.7-95.1]	<0.001			0.694
		IDF2005	7	4081	563	14.5 [11.0-18.4]	3.06 [2.26-4.13]	89.3 [80.5-94.1]	<0.001			0.620
Study size (median, 292)	Overall									0.002		
		ATPIII2001	38	16984	3179	16.7 [14.6-18.8]	3.56 [3.18-3.99]	92.1 [90.1-93.7]	<0.001		0.115	0.040
		ATPIII2004-2005	20	11255	2768	24.6 [20.6-28.8]	4.90 [4.30-5.60]	95.8 [94.6-96.8]	<0.001		0.989	0.870
		IDF2005	16	8250	1471	18.0 [14.0-22.4]	4.90 [4.20-5.60]	95.8 [94.4-96.9]	<0.001		0.388	0.610
	Above median									0.095		
		ATPIII2001	17	13382	2646	18.3 [15.3-21.4]	4.60 [3.98-5.33]	95.3 [93.7-96.5]	<0.001			0.012
		ATPIII2004-2005	12	10176	2517	24.7 [19.7-29.9]	5.96 [5.13-6.92]	97.2 [96.2-97.9]	<0.001			0.849
		IDF2005	10	7264	1312	19.0 [13.6-25.0]	6.20 [5.28-7.29]	97.4 [96.4-98.1]	<0.001			0.354
	Below median									0.059		
		ATPIII2001	21	3602	533	15.1 [12.8-17.6]	2.02 [1.64-2.50]	75.5 [62.7-83.9]	<0.001			0.137
		ATPIII2004-2005	8	1079	251	25.6 [17.1-32.9]	3.03 [2.29-4.01]	89.1 [80.8-93.8]	<0.001			0.217
		IDF2005	6	986	159	16.1 [12.7-19.8]	1.48 [1.00-2.34]	54.5 [00.0-81.8]	0.052			0.817
Age, median 40.9 years	Overall									0.064		
		ATPIII2001	32	13234	2343	16.6 [14.4-18.9]	3.44 [3.04-3.90]	91.6 [89.2-93.4]	<0.001		0.000	0.373

		ATPIII2004-2005	13	7112	1829	23.8 [18.2-30.0]	5.72 [4.94-6.63]	96.9 [95.9-97.7]	<0.001		0.479	0.580
		IDF2005	14	5818	1112	18.5 [13.5-24.1]	5.14 [4.42-5.98]	96.2 [94.9-97.2]	<0.001		0.361	0.850
	Above median									0.525		
		ATPIII2001	16	8392	1757	19.7 [17.1-22.5]	2.98 [2.45-3.62]	88.7 [83.3-92.4]	<0.001			0.311
		ATPIII2004-2005	6	2204	659	26.6 [15.0-40.0]	6.51 [5.28-8.02]	97.6 [96.4-98.4]	<0.001			0.580
		IDF2005	5	1891	474	22.3 [11.5-35.5]	6.40 [5.06-8.10]	97.6 [96.1-98.5]	<0.001			0.500
	Below median									0.021		
		ATPIII2001	16	4842	586	13.2 [11.2-15.2]	1.95 [1.52-2.50]	73.7 [56.8-83.9]	<0.001			0.011
		ATPIII2004-2005	7	4908	1170	21.5 [15.6-28.2]	5.12 [4.10-6.39]	96.2 [94.0-97.5]	<0.001			0.485
		IDF2005	9	3927	638	16.4 [11.8-21.6]	3.94 [3.15-4.94]	93.6 [89.9-95.9]	<0.001			0.813
Proportion of male, median 70.7%	Overall									0.005		
		ATPIII2001	37	16887	3149	16.4 [14.4-18.6]	3.58 [3.20-4.01]	92.2 [90.2-93.8]	<0.001		0.148	0.021
		ATPIII2004-2005	18	9397	2163	24.1 [20.0-28.5]	4.69 [4.08-5.40]	95.5 [94.0-96.6]	<0.001		0.574	0.442
		IDF2005	15	6606	1226	18.2 [13.7-23.3]	5.00 [4.31-5.80]	96.0 [94.6-97.0]	<0.001		0.002	0.773
	Above median									0.000		
		ATPIII2001	19	7655	1283	14.9 [12.0-18.0]	3.57 [3.04-4.19]	92.1 [89.2-94.3]	<0.001			0.269
		ATPIII2004-2005	7	4454	939	22.6 [18.5-26.9]	2.83 [2.07-3.88]	87.5 [76.6-93.3]	<0.001			0.476
		IDF2005	6	2806	319	12.2 [09.3-15.3]	2.23 [1.51-3.29]	79.9 [56.3-90.7]	<0.001			0.328
	Below median									0.112		
		ATPIII2001	18	9232	1866	18.0 [15.2-20.9]	3.39 [2.85-4.02]	91.3 [87.7-93.8]	<0.001			0.068
		ATPIII2004-2005	11	4943	1224	24.9 [18.3-32.2]	5.59 [4.75-6.58]	96.8 [95.6-97.7]	<0.001			0.773
		IDF2005	9	3800	907	22.3 [16.5-28.7]	4.38 [3.55-5.42]	94.8 [92.0-96.6]	<0.001			0.730
Proportion of smokers, median 39.8%	Overall									0.003		
		ATPIII2001	30	14607	2694	16.4 [14.1-18.8]	3.69 [3.26-4.18]	92.7 [90.6-94.3]	<0.001		0.234	0.059
		ATPIII2004-2005	15	9087	2344	25.7 [20.8-31.0]	5.41 [4.70-6.24]	96.6 [95.5-97.4]	<0.001		0.193	0.922
		IDF2005	11	4527	707	16.3 [11.3-22.0]	4.87 [4.08-5.82]	95.8 [94.0-97.0]	<0.001		0.565	0.314
	Above median									0.001		
		ATPIII2001	14	4546	695	14.8 [11.7-18.2]	2.81 [2.27-3.50]	87.4 [80.5-91.8]	<0.001			0.863

		ATPIII2004-2005	8	6211	1750	28.8 [21.9-36.2]	5.86 [4.85-7.08]	97.1 [95.8-98.0]	<0.001			0.676
		IDF2005	5	1917	331	18.4 [07.5-32.8]	7.00 [5.66-8.74]	98.0 [96.8-98.7]	<0.001			0.456
	Below median									0.140		
		ATPIII2001	16	10061	1999	17.6 [14.5-20.8]	4.05 [3.40-4.76]	93.9 [91.5-95.6]	<0.001			0.057
		ATPIII2004-2005	7	2876	594	22.2 [15.9-29.3]	4.22 [3.29-5.42]	94.4 [90.8-96.6]	<0.001			0.557
		IDF2005	6	2610	376	14.6 [10.8-18.8]	2.79 [1.97-3.94]	87.1 [74.4-93.6]	<0.001			0.723
Antiretroviral (ART) use	Overall									0.130		
		ATPIII2001	37	14807	2780	15.5 [13.4-17.7]	3.26 [2.89-3.68]	90.6 [88.0-92.6]	<0.001		0.001	0.016
		ATPIII2004-2005	12	4939	1050	21.2 [15.9-27.0]	4.27 [3.55-5.13]	94.5 [92.1-96.2]	<0.001		0.710	0.850
		IDF2005	18	6601	1178	17.8 [13.6-22.4]	4.48 [3.88-5.18]	95.0 [93.4-96.3]	<0.001		0.322	0.586
	Non-ART									0.000		
		ATPIII2001	17	2659	319	11.8 [09.3-14.7]	1.96 [1.54-2.48]	73.9 [57.8-83.8]	<0.001			0.710
		ATPIII2004-2005	4	2508	499	19.9 [18.3-21.5]	1.00 [1.00-2.54]	00.0 [00.0-84.5]	0.398			0.980
		IDF2005	7	1628	216	14.9 [08.6-22.6]	3.45 [2.60-4.57]	91.6 [85.3-95.2]	<0.001			0.351
	On ART									0.731		
		ATPIII2001	20	12148	2461	18.4 [15.9-21.1]	3.27 [2.77-3.85]	90.6 [87.0-93.3]	<0.001			0.240
		ATPIII2004-2005	8	2431	551	21.6 [13.5-31.0]	5.27 [4.31-6.46]	96.4 [94.6-97.6]	<0.001			0.999
		IDF2005	11	4973	962	19.6 [14.2-25.6]	4.83 [4.04-5.78]	95.7 [93.9-97.0]	<0.001			0.634
Severity of HIV-infection, median CD4 394	Overall									0.015		
		ATPIII2001	24	9402	1709	16.4 [13.8-19.2]	3.46 [2.99-4.00]	91.6 [88.8-93.7]	<0.001		0.514	0.162
		ATPIII2004-2005	10	4478	1272	25.2 [19.0-32.0]	4.67 [3.86-5.67]	95.4 [93.3-96.9]	<0.001		0.747	0.298
		IDF2005	7	1386	199	14.9 [11.5-18.7]	1.82 [1.23-2.70]	70.0 [34.2-86.3]	0.003		0.001	0.292
	Above median									0.000		
		ATPIII2001	10	5888	1156	17.4 [13.1-22.1]	4.59 [3.78-5.58]	95.3 [93.0-96.8]	<0.001			0.202
		ATPIII2004-2005	7	3719	1088	24.6 [16.6-33.6]	5.53 [4.47-6.83]	96.7 [95.0-97.9]	<0.001			0.269
		IDF2005	2	632	66	10.4 [08.2-12.9]	1.00 [-]	00.0 [-]	0.635			-
	Below median									0.020		
		ATPIII2001	14	3514	553	15.6 [12.9-18.5]	2.22 [1.73-2.84]	79.7 [66.7-87.6]	<0.001			0.921
		ATPIII2004-2005	3	759	184	26.5 [19.3-34.5]	1.83 [1.00-3.39]	70.2 [00.0-91.3]	0.035			0.484

		IDF2005	5	754	133	17.5 [14.4-20.8]	1.13 [1.00-1.76]	22.3 [00.0-67.8]	0.272			0.830
HIV duration, median 67.5 months	Overall									0.240		
		ATPIII2001	13	3713	632	17.5 [13.3-22.2]	3.44 [2.81-4.21]	91.6 [87.4-94.3]	<0.001		0.044	0.562
		ATPIII2004-2005	6	2482	506	24.7 [17.8-32.3]	3.80 [2.84-5.08]	93.1 [87.6-96.1]	<0.001		0.251	0.170
		IDF2005	2	1399	298	19.1 [12.7-26.4]	2.15 [-]	78.3 [-]	0.032		-	-
	Above median									0.338		
		ATPIII2001	7	2735	502	20.6 [13.8-28.4]	4.61 [3.64-5.84]	95.3 [92.4-97.1]	<0.001			0.336
		ATPIII2004-2005	3	393	115	32.0 [11.8-56.7]	4.98 [3.40-7.30]	96.0 [91.3-98.1]	<0.001			0.141
		IDF2005	-									
	Below median									0.007		
		ATPIII2001	6	978	130	13.2 [11.2-15.4]	1.00 [1.00-1.73]	00.0 [00.0-66.5]	0.581			0.265
		ATPIII2004-2005	3	2089	391	19.1 [15.9-22.6]	1.80 [1.00-3.33]	69.1 [00.0-91.0]	0.039			0.707
		IDF2005	2	1399	298	19.1 [12.7-26.4]	2.15 [-]	78.3 [-]	0.032			-
ART duration, median 27 months	Overall									0.352		
		ATPIII2001	16	8193	1503	16.3 [13.3-19.6]	3.82 [3.22-4.52]	93.1 [90.4-95.1]	<0.001		0.649	0.102
		ATPIII2004-2005	4	1447	246	22.1 [12.2-34.0]	4.80 [3.48-6.63]	95.7 [91.7-97.7]	<0.001		0.192	0.112
		IDF2005	6	2054	288	14.0 [09.9-18.8]	2.84 [2.02-4.00]	87.6 [75.4-93.7]	<0.001		0.811	0.765
	Above median									0.544		
		ATPIII2001	7	4457	846	17.1 [12.1-22.9]	4.82 [3.83-6.07]	95.7 [93.2-97.3]	<0.001			0.336
		ATPIII2004-2005	3	769	150	25.6 [09.7-45.8]	5.67 [3.98-8.07]	96.9 [93.7-98.5]	<0.001			0.031
		IDF2005	3	1006	155	14.6 [07.0-24.2]	3.93 [2.54-6.10]	93.5 [84.5-97.3]	<0.001			0.854
	Below median									0.740		
		ATPIII2001	9	3736	657	15.6 [12.0-19.6]	3.10 [2.39-4.02]	89.6 [82.5-93.8]	<0.001			0.187
		ATPIII2004-2005	1	678	96	14.2 [11.6-17.0]	-	-	-			-
		IDF2005	3	1048	133	13.4 [09.1-18.3]	1.88 [1.02-3.46]	71.7 [40.0-91.6]	0.030			0.701
Proportion on ART, median 76.15%	Overall									0.006		
		ATPIII2001	19	7817	1385	16.9 [14.3-19.7]	3.10 [2.60-3.69]	89.6 [85.2-92.7]	<0.001		0.172	0.572

		ATPIII2004-2005	10	7041	1838	26.2 [20.9-31.9]	5.10 [4.25-6.12]	96.2 [94.5-97.3]	<0.001		0.256	0.981
		IDF2005	7	3760	662	17.1 [13.1-21.6]	3.34 [2.51-4.45]	91.1 [84.1-95.0]	<0.001		0.176	0.822
	Above median									0.111		
		ATPIII2001	12	5700	967	15.8 [12.4-19.6]	3.64 [2.97-4.46]	92.5 [88.7-95.0]	<0.001			0.496
		ATPIII2004-2005	2	903	262	38.3 [15.5-64.3]	5.07 [-]	96.1 [-]	<0.001			-
		IDF2005	3	1311	190	14.4 [11.6-17.3]	1.38 [1.0-2.56]	47.7 [00.0-84.7]	0.148			0.880
	Below median									0.385		
		ATPIII2001	7	2117	418	19.1 [16.2-22.3]	1.68 [1.12-2.52]	64.4 [19.8-84.2]	0.009			0.507
		ATPIII2004-2005	8	6138	1576	23.8 [18.0-30.0]	5.40 [4.42-6.58]	96.6 [94.9-97.7]	<0.001			0.504
		IDF2005	4	2449	472	19.4 [12.9-26.9]	4.12 [2.89-5.86]	94.1 [88.0-97.1]	<0.001			0.947
Proportion on NNRTIs, median 43.4%	Overall									0.142		
		ATPIII2001	17	7953	1518	17.6 [14.3-21.1]	3.90 [3.32-4.59]	93.4 [90.9-95.2]	<0.001		0.221	0.434
		ATPIII2004-2005	7	4071	1087	26.2 [17.9-35.5]	6.48 [5.35-7.85]	97.6 [96.5-98.4]	<0.001		0.020	0.976
		IDF2005	6	4087	647	16.4 [10.1-23.8]	5.72 [4.56-7.18]	96.9 [95.2-98.1]	<0.001		0.058	0.683
	Above median									0.001		
		ATPIII2001	10	4745	993	19.5 [15.5-23.9]	3.48 [2.76-4.37]	91.7 [86.9-94.8]	<0.001			0.533
		ATPIII2004-2005	3	1881	355	17.2 [09.5-26.6]	4.94 [3.36-7.25]	95.9 [91.2-98.1]	<0.001			0.464
		IDF2005	2	1078	113	10.5 [08.7-12.4]	1.00 [-]	00.0 [-]	0.690			-
	Below median									0.008		
		ATPIII2001	7	3208	525	15.1 [10.1-21.0]	4.19 [3.26-5.39]	94.3 [90.6-96.6]	<0.001			0.743
		ATPIII2004-2005	4	2190	732	33.8 [23.4-45.1]	5.40 [4.00-7.29]	96.6 [93.7-98.1]	<0.001			0.848
		IDF2005	4	3009	534	19.8 [10.4-31.2]	6.67 [5.12-8.68]	97.8 [96.2-98.7]	<0.001			0.570
Proportion on NRTIs, median 76.95%	Overall									0.303		
		ATPIII2001	5	1781	361	19.7 [13.1-27.2]	3.44 [2.45-4.85]	91.6 [83.3-95.7]	<0.001		0.474	0.930
		ATPIII2004-2005	2	1148	390	38.7 [15.6-64.9]	8.40 [-]	98.6 [-]	<0.001		-	-
		IDF2005	2	1133	263	27.6 [05.3-58.8]	10.13 [-]	99.0 [-]	<0.001		-	-
	Above median									0.268		

		ATPIII2001	3	1107	189	17.9 [09.4-28.7]	3.43 [2.14-5.49]	91.5 [78.2-96.7]	<0.001			0.900
		ATPIII2004-2005	2	1148	390	38.7 [15.6-64.9]	8.40 [-]	98.6 [-]	<0.001			-
		IDF2005	2	1133	263	27.6 [05.3-58.8]	10.13 [-]	99.0 [-]	<0.001			-
	Below median									-		
		ATPIII2001	2	674	172	22.8 [14.3-32.7]	2.17 [-]	78.8 [-]	<0.001			-
		ATPIII2004-2005	0	-	-	-	-	-	-			-
		IDF2005	0	-	-	-	-	-	-			-
Proportion on PI, median 37.35%	Overall									0.022		
		ATPIII2001	19	8752	1668	18.2 [15.1-21.4]	3.76 [3.21-4.39]	92.9 [90.3-94.8]	<0.001		0.593	0.647
		ATPIII2004-2005	10	7136	1924	28.0 [21.3-35.3]	6.51 [5.56-7.61]	97.6 [96.8-98.3]	<0.001		0.517	0.737
		IDF2005	7	5327	921	17.2 [11.6-23.5]	5.67 [4.61-6.99]	96.9 [95.3-98.0]	<0.001		0.016	0.878
	Above median									0.353		
		ATPIII2001	11	4869	878	17.7 [12.9-23.0]	4.53 [3.76-5.46]	95.1 [92.9-96.6]	<0.001			0.960
		ATPIII2004-2005	5	3543	813	25.8 [16.2-36.7]	6.99 [5.60-8.73]	98.0 [96.8-98.7]	<0.001			0.164
		IDF2005	6	4927	881	18.5 [12.3-25.6]	5.93 [4.75-7.40]	97.2 [95.6-98.2]	<0.001			0.679
	Below median									0.000		
		ATPIII2001	8	3883	790	19.3 [16.3-22.6]	2.30 [1.66-3.18]	81.0 [63.5-90.1]	<0.001			0.411
		ATPIII2004-2005	5	3593	1111	30.4 [21.6-39.9]	5.56 [4.30-7.19]	96.8 [94.6-98.1]	<0.001			0.783
		IDF2005	1	400	40	10.0 [07.2-13.4]	-	-	-			-

- not computable; ATP, Adults Treatment Panel; ART, antiretroviral; CI, confidence interval; IDF, International Diabetes Federation; HIV, human immunodeficiency virus; NNRTI, non-nucleoside reverse transcriptase inhibitors; NRTI, nucleoside reverse transcriptase; PI, protease inhibitors
